# A Change in C–H Activation Mechanism: Experimental and Computational Investigations of Rh-Catalyzed Disubstituted Benzene Functionalization

**DOI:** 10.1021/acs.organomet.5c00379

**Published:** 2025-10-29

**Authors:** Christopher W. Reid, Chi Zhang, Lauren E. Baptiste, K. N. Houk, William A. Goddard III, T. Brent Gunnoe

**Affiliations:** † Department of Chemistry, 2358 University of Virginia, Charlottesville, Virginia 22904, United States; ‡ Materials and Process Simulation Center, 6469 California Institute of Technology, Pasadena, California 91125, United States; § Department of Chemistry and Biochemistry, University of California, Los Angeles, California 90095-1569, United States

## Abstract

We report the ethenylation of 1,3- and 1,2-disubstituted benzenes using [(η^2^-C_2_H_4_)_2_Rh­(μ-OAc)]_2_ as a catalyst precursor and Cu­(OPiv)_2_ as the oxidant. The regioselectivity of alkenylation for 1,3-disubstituted benzenes produces 3,5-disubstituted styrene products, while the alkenylation of 1,2-disubstituted benzenes produces 3,4-disubstituted styrene products. The rate of alkenylation is influenced by steric and electronic factors based on the substituents of the benzene unit. In all cases, 1,2-disubstituted benzenes react faster than 1,3-disubstituted benzenes, with a rate difference that is from 2 times up to >70 times more rapid for 1,2-disubstituted substrates. This is likely due to the difference in the number of accessible C–H bonds based on the steric protection of C–H bonds adjacent to functionality. Furthermore, the rate of alkenylation is influenced by the arene substituent electronics. The rates of alkenylation for 1,2-disubstituted benzenes follow the trend OMe > Me > CF_3_ > Cl, while for 1,3-disubstituted benzenes the trend is CF_3_ > Cl > Me > OMe. Using quantum mechanics DFT calculations, we found that the C–H activation step can occur by two different mechanisms. The electronic properties of substituents on the arene ring change the preferred C–H bond-breaking mechanism for 1,2-disubstituted and 1,3-disubstituted benzenes.

## Graphical abstract

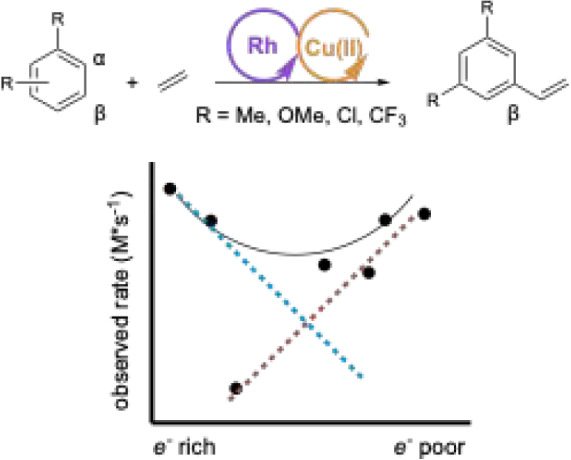

## Introduction

Reactions that form C–C bonds have emerged as key reactions in all stages of organic synthesis. For example, due to their high efficiency and versatility, classic Pd-catalyzed cross-coupling reactions are heavily used for the formation of C–C bonds.^1^ Many variations of cross-coupling reactions have been developed for which a catalyst, ligand, and a prefunctionalized substrate (often a halogenated substrate) react with a stoichiometric amount of a coupling partner to form a C–C coupled product along with stoichiometric waste (Scheme 1).^2–5^ A potentially more desirable strategy for forming C–C bonds is through C–H functionalization (i.e., reactions that do not require a prefunctionalized substrate).

**Scheme 1:**
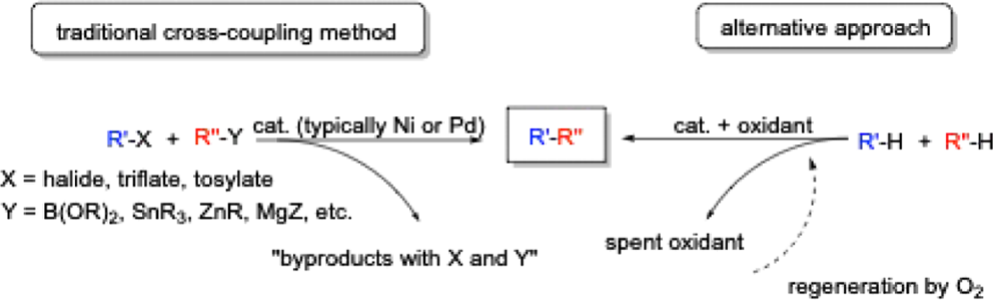
Methods to Form C–C bonds Showing Traditional Cross Coupling Methods and an Alternative Approach That Directly Functionalizes C–H Bonds

C–H functionalization reactions are sometimes categorized into two primary types: *directed and nondirected* C–H functionalization (Scheme 2).^6^ *Directed* C–H functionalization involves the use of a halogen or heteroatomic directing group (DG) covalently attached to a substrate to promote direct C–H activation. In contrast, *nondirected* C–H functionalization occurs in the absence of a directing group on the substrate.

**Scheme 2:**
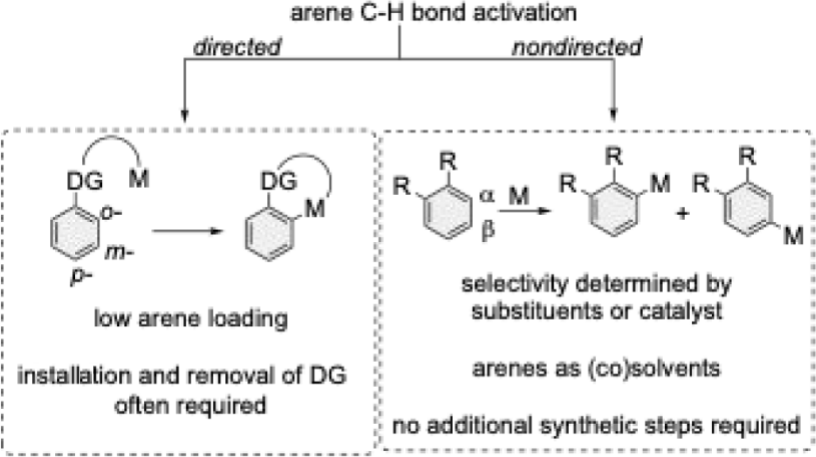
Differences between Directed and Nondirected C–H Activation of Arenes

A challenge with nondirected C–H alkenylation is that large arene loadings (often used neat or as a cosolvent) are often needed to observe reactivity, and the regioselectivity is often poor.^7^ An early example of nondirected C–H alkenylation is the Pd-catalyzed alkenylation of arenes using a Cu­(II) oxidant, known as the Fujiwara-Moritani reaction.^8^ Since then, additional examples of Pd-catalyzed nondirected C–H alkenylations have been reported.^9^ Also, examples using Rh catalyst precursors have been developed for nondirected C–H alkenylation.^10,11^

Several reports of directed alkenylation of symmetric 1,3- and 1,2-disubstituted arenes have emerged (Schemes 3 and 4). The Ackermann group observed a mixture of β- and α-alkenylated products when using Pd­(OAc)_2_ with a sulfur-based ligand across 1,2- and 1,3-disubstituted benzenes.^12^ The Sanford group reported on the use of Pd­(OAc)_2_/3,5-dichloropyridine to catalyze the alkenylation of *o*-xylene and *o*-dichlorobenzene, observing a mix of β- and α-alkenylated products.^13^ Changing the ligand from 3,5-dichloropyridine to acridine was observed to enhance the regioselectivity from ∼3:1 to ∼5:1 (β:α) using *o*-xylene as the arene and ethyl acrylate as the olefin. Reports from the Joo group on Pd­(OAc)_2_-catalyzed disubstituted arene alkenylation using a series of pyrazolopyridone ligands revealed that the regioselectivity is dependent on the electronics of the ligand used.^14^ For electron-rich arenes (e.g., disubstituted anisole derivatives), primarily, *meta* alkenylation was observed. The Glorius group reported on the Rh-catalyzed alkenylation of bromoarenes, where 1,2-disubstituted benzenes primarily gave the β-alkenylated product, and 1,3-disubstituted benzenes gave the 1,3,5-trisubstituted product.^15^ Yu and coworkers studied the alkenylation of both electron-rich and electron-poor disubstituted arenes, where β-selectivity was displayed for a range of 1,2-disubstituted substrates.^16^ Our group has studied the alkenylation of benzene with substituted styrenes for the synthesis of stilbene derivatives, for which the alkenylation of disubstituted benzenes occurs at the most sterically accessible β-position.^17^ To the best of our knowledge, examples of disubstituted benzene alkenylation using ethylene as the olefin have not been reported.

**Scheme 3:**
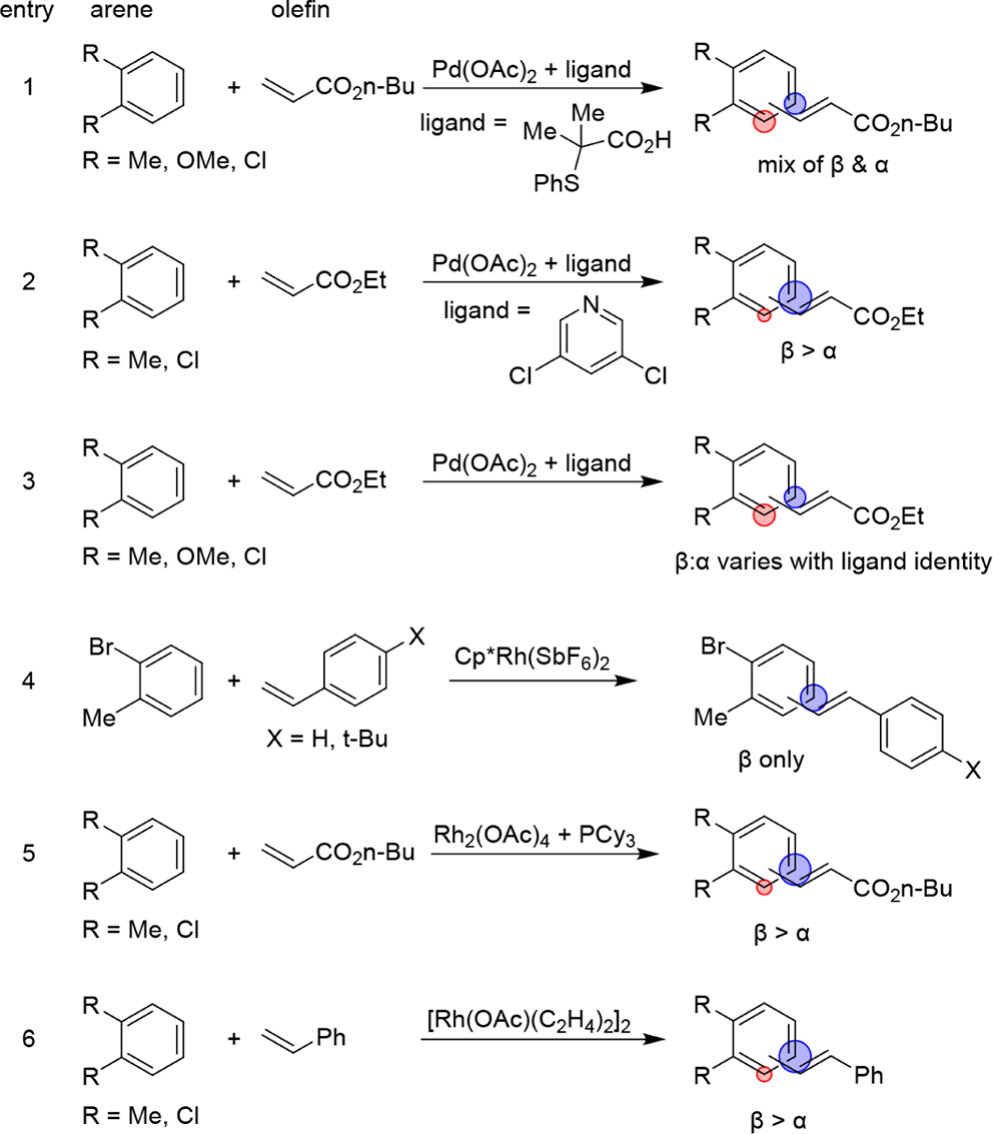
Reported Methods for the Alkenylation of 1,2-disubstituted Benzenes

**Scheme 4:**
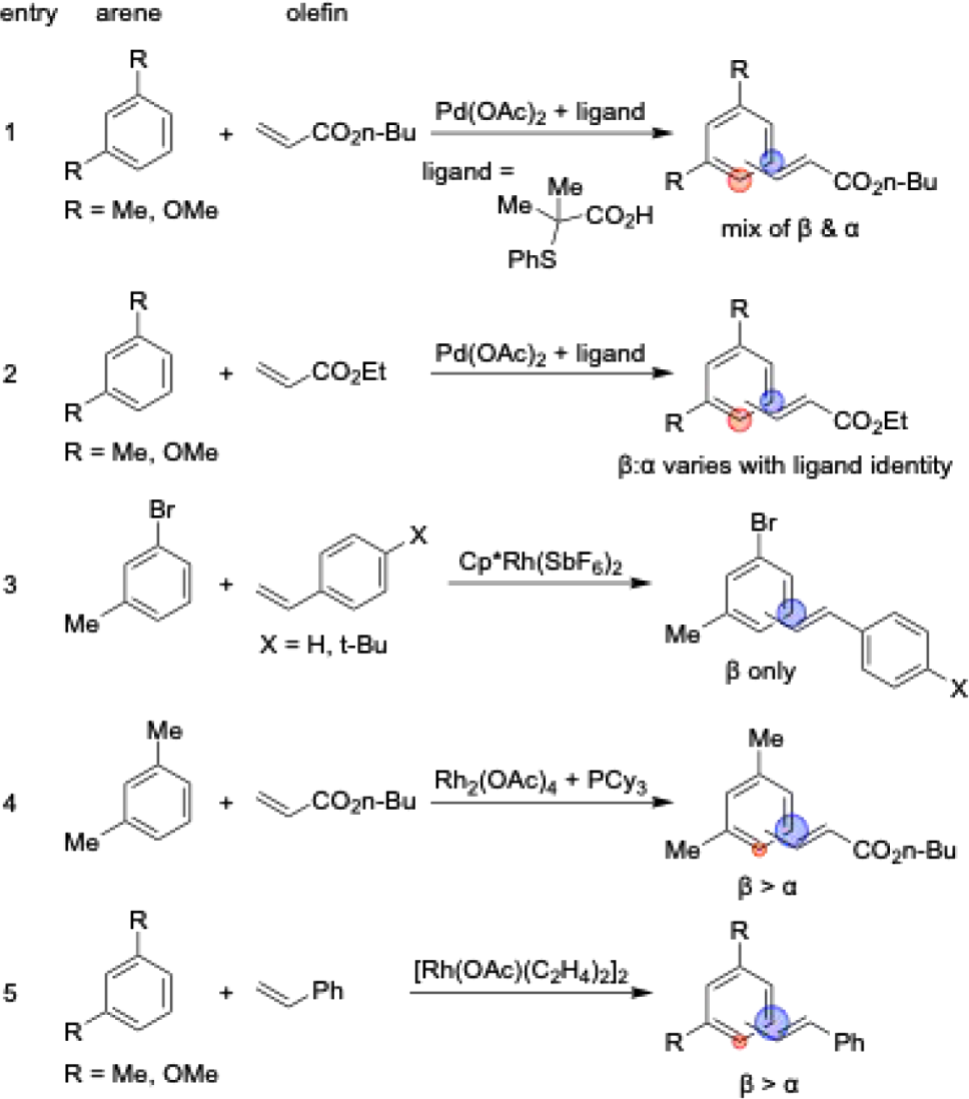
Reported Methods for the Alkenylation of 1,3-disubstituted Benzenes

Arene alkenylation catalysis using benzene or substituted benzenes and ethylene to produce alkenyl arenes has been reported using Ru,^18–28^ Pt,^29–35^ Ir,^36^ Pd,^37–39^ and Rh.^17,38,40–53^ We reported that [(η^2^-C_2_H_4_)_2_Rh­(μ-OAc)]_2_ is a catalyst precursor for the conversion of arenes (e.g., benzene and monosubstituted benzenes) and olefins (e.g., ethylene, α-olefins, and multisubstituted olefins) directly to alkenyl arenes. We have proposed that this Rh-based arene alkenylation catalyst operates through arene C–H activation, olefin insertion into Rh–aryl bonds, and β-hydride elimination to produce the alkenyl arene. The Rh–H intermediates then react with 2 eq. of Cu­(II) carboxylate salts to regenerate the active catalyst (Scheme 5).

**Scheme 5:**
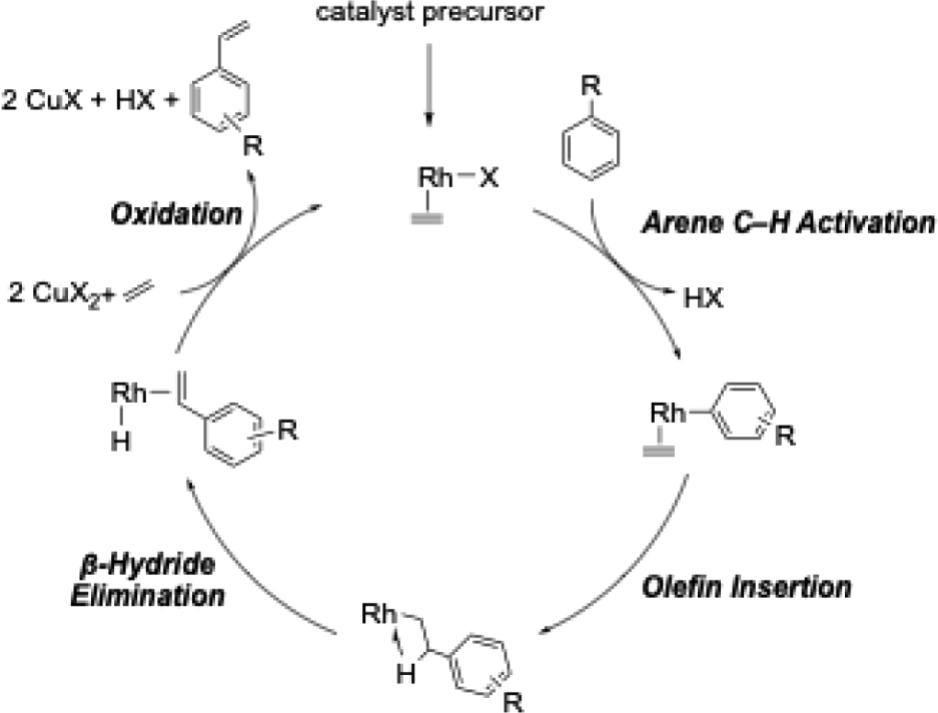
Proposed Mechanism for Rh-catalyzed Arene Alkenylation (X = OAc, OPiv, or OHex)

Herein, we extend previously reported arene alkenylation reactions to symmetric disubstituted benzenes and present both experimental and computational results. We propose that for a set of relatively simple arene substrates, the same catalyst undergoes two different C–H activation mechanisms. To our knowledge, evidence for a change in C–H activation mechanism for the same catalyst system has been sparsely reported.^54,55^

## Results and Discussion

Heating a solution of neat *m*-xylene (*m*-Me) with [(η^2^-C_2_H_4_)_2_Rh­(μ-OAc)]_2_ (**1**) as the catalyst precursor (0.001 mol % relative to *m*-xylene) with 480 eq. Cu­(OPiv)_2_ (relative to **1**), 960 eq. HOPiv (relative to **1**), and 50 psig ethylene at 150 °C yielded 3 (1) TOs of 3,5-dimethylstyrene as the only product after 1 h (Table 1, entry 1). Under the same reaction conditions as *m*-Me, reactions in neat 1,3-dimethoxybenzene (*m*-OMe) generated <1 TO after 1 h (Table 1, entry 2). The alkenylation of 1,3-dichlorobenzene (*m*-Cl) yielded 11 TOs of 3,5-dichlorostyrene as the sole product after heating at 150 °C for 1 h (Table 1, entry 3). Reactions in neat 1,3-bis­(trifluoromethyl)­benzene (*m*-CF_3_) gave 18 (3) TOs of 3,5-bis­(trifluoromethyl)­styrene with quantitative selectivity (Table 1, entry 4). For the 1,3-disubstituted benzenes tested (Table 1), using the TO data after 1 h, we observed a trend showing that electron-poor benzenes react faster than electron-rich benzenes (i.e., *m*-CF_3_ > *m*-Cl > *m*-Me > *m*-OMe).

**Table 1:**
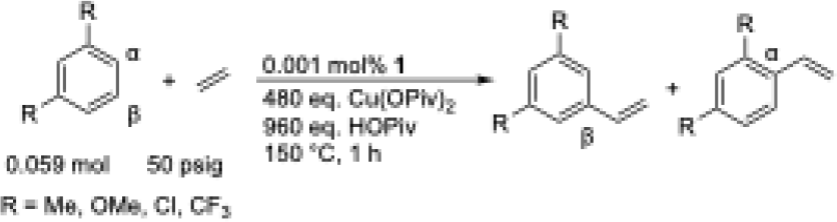
Ethenylation of Symmetric 1,3-Disubstituted Benzenes

Entry	R	Total TOs^a^	Ratio α:β^a^
1	Me	3(1)	0:100
2	OMe	<1	-
3	CI	11	0:100
4	CF_3_	18(3)	0:100

a TOs and regioselectivity ratios determined by GC-FID. Reaction conditions: 0.059 mol arene, 0.001 mol % [(η^2^-C_2_H_4_)_2_Rh­(μ-OAc)]_2_ (relative to arene), 480 eq. Cu­(OPiv)_2_ (relative to a single Rh atom), 960 eq. HOPiv (relative to a single Rh atom), 50 psig ethylene, 150 °C, 1 h. Quantified using GC-FID analysis with HMB used as an internal standard. Numbers in parentheses represent the standard deviation based on a minimum of three independent experiments.

We tested the alkenylation of 1,2-disubstituted benzenes under the same reaction conditions as those for the 1,3-disubstituted benzenes (Table 2). Heating a solution of neat *o*-xylene (*o*-Me) at 150 °C yielded 44 (3) TOs of 3,4-dimethylstyrene as the only product after 1 h (Table 2, entry 1). Conversely to the results observed for 1,3-disubstituted benzenes, 1,2-dimethoxybenzene (*o*-OMe) yielded 68 (12) TOs of 3,4-dimethoxystyrene as the sole product after 1 h at 150 °C (Table 2, entry 2). The ethenylation of 1,2-dichlorobenzene (*o*-Cl) yielded 20 (3) TOs of 3,4-dichlorostyrene after heating at 150 °C for 1 h (Table 2, entry 3). Reactions in neat 1,3-bis­(trifluoromethyl)­benzene (*o*-CF_3_) gave 31 (3) TOs of 3,4-bis­(trifluoromethyl)­styrene with quantitative selectivity (Table 2, entry 4). Similar to the alkenylation of 1,3-disubstituted benzenes, the β-alkenylated product is observed with quantitative selectivity for all substrates. In contrast to the results of 1,3-disubstituted benzenes, we observed a trend that for 1,2-disubstituted benzenes, electron-rich benzenes react faster than electron-poor benzenes (i.e., *o*-OMe > *o*-Me > *o*-CF_3_ > *o*-Cl).

**Table 2:**
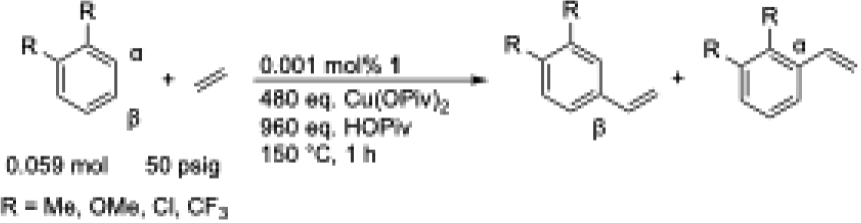
Ethenylation of Symmetric 1,2-Disubstituted Benzenes

Entry	R	Total TOs^a^	Ratio α:β^a^
1	Me	44(3)	0:100
2	OMe	68(12)	0:100
3	CI	20(3)	0:100
4	CF_3_	31(3)	0:100

a TOs and regioselectivity ratios determined by GC-FID. Reaction conditions: 0.059 mol arene, 0.001 mol % [(η^2^-C_2_H_4_)_2_Rh­(μ-OAc)]_2_ (relative to arene), 480 eq. Cu­(OPiv)_2_ (relative to a single Rh atom), 960 eq. HOPiv (relative to a single Rh atom), 50 psig ethylene, 150 °C, 1 h. Quantified using GC-FID analysis with HMB used as an internal standard. Numbers in parentheses represent the standard deviation based on a minimum of three independent experiments.

We compared the rates of ethenylation for both 1,3- and 1,2-disubstituted benzenes (Figures 1 and 2). Figure 1 shows that the rate of ethenylation for 1,3-disubstituted benzenes (for which R = CF_3_, Cl, Me, and OMe) follows the trend *m*-CF_3_ > *m*-Cl > *m*-Me > *m*-OMe. Figure 2 shows that the rate of ethenylation for 1,2-disubstituted benzenes (for which R = CF_3_, Cl, Me, and OMe) follows the trend *o*-OMe > *o*-Me > *o*-CF_3_ > *o*-Cl. These results show that electron-rich 1,2-disubstituted benzenes (i.e., *o*-OMe, *o*-Me) react faster than electron-poor 1,2-disubstituted benzenes (i.e., *o*-Cl, *o*-CF_3_) with a rate difference of 3.4 between the fastest (*o*-OMe) and the slowest (*o*-Cl). The opposite is true for 1,3-disubstituted benzenes; that is, electron-poor 1,3-disubstituted benzenes (i.e., *m*-Cl, *m* -CF_3_) react more rapidly than electron-rich 1,3-disubstituted benzenes (i.e., *m*-OMe, *m*-Me) with a rate difference of >18 between the fastest (*m*-CF_3_) and the slowest (*m*-OMe; note that a rate was not determined since the product could not be quantified). Steric effects likely play a role when comparing the rates of ethenylation for 1,2- and 1,3-disubstituted benzenes (see next paragraph).

**Figure 1:**
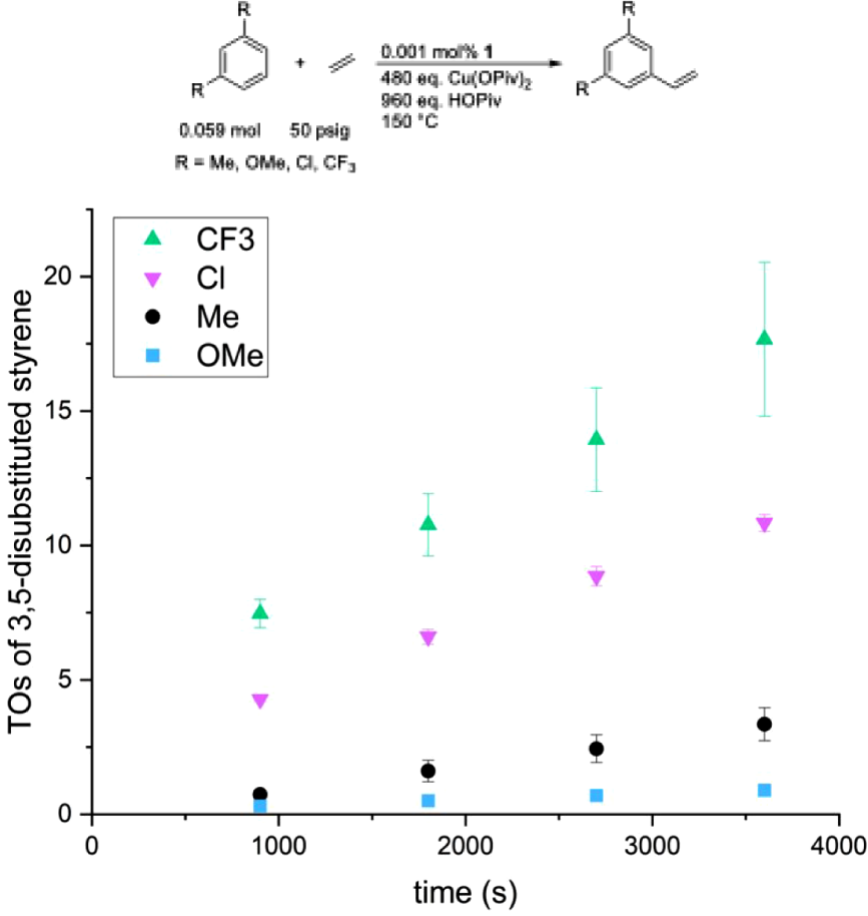
Turnovers (TOs) versus time plot for symmetric 1,3-disubstituted benzenes alkenylation. Reaction conditions: 0.059 mol of arene, 0.001 mol % [(η^2^-C_2_H_4_)_2_Rh­(μ-OAc)]_2_ (relative to arene), 480 eq. Cu­(OPiv)_2_ (relative to a single Rh atom), 960 eq. HOPiv (relative to a single Rh atom), 50 psig ethylene, 150 °C. Quantified using GC-FID analysis with HMB used as an internal standard. Error bars represent the standard deviation based on a minimum of three independent experiments.

**Figure 2:**
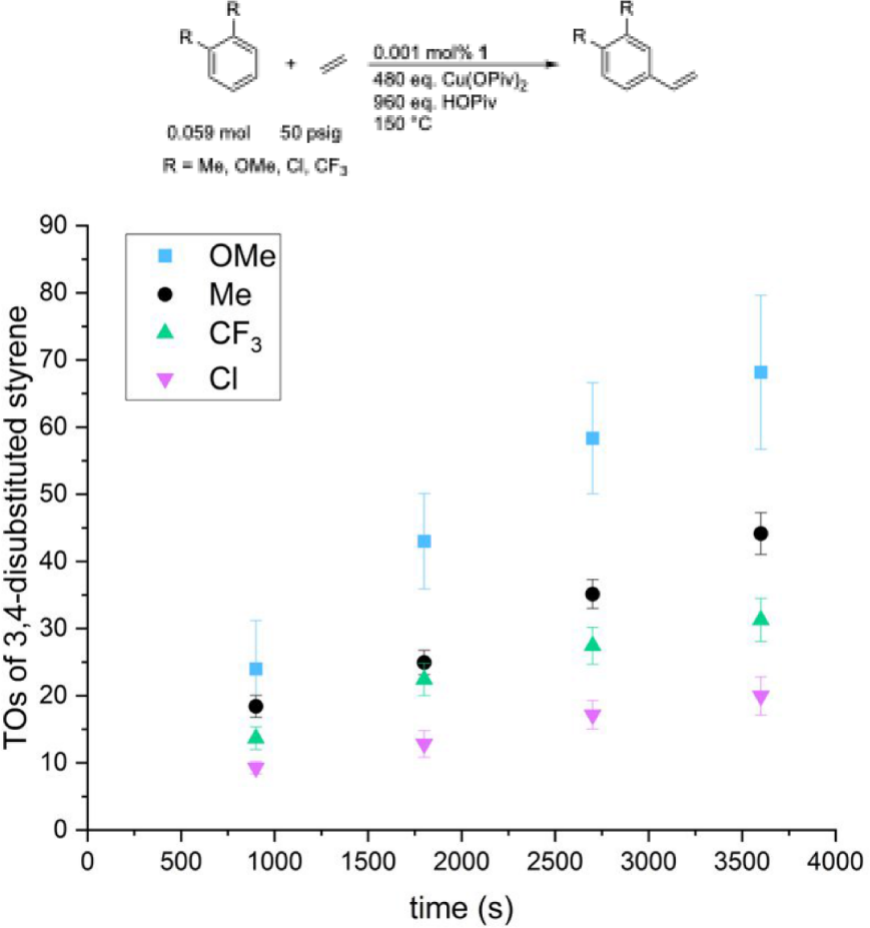
TOs versus time plot for symmetric 1,2-disubstituted benzenes alkenylation. Reaction conditions: 0.059 mol of arene, 0.001 mol % [(η^2^-C_2_H_4_)_2_Rh­(μ-OAc)]_2_ (relative to arene), 480 eq. Cu­(OPiv)_2_ (relative to a single Rh atom), 960 eq. HOPiv (relative to a a single Rh atom), 50 psig ethylene, 150 °C. Quantified using GC-FID analysis with HMB used as an internal standard. Error bars represent the standard deviation based on a minimum of three independent experiments.

As for the steric effect, comparing 1,3- to 1,2-disubstituted benzenes for which the substituents are the same (e.g., *m*-Me versus *o*-Me) shows an increase in the rate of alkenylation for 1,2-disubstituted benzenes. The chlorinated arenes show the least difference in rate, with *o*-Cl reacting approximately twice as fast as *m*-Cl, and OMe-substituted arenes show the greatest difference by a factor of at least 70. If C–H bonds adjacent to substituents are considered inaccessible, the 1,2-disubstituted benzenes have two accessible C–H bonds, while the 1,3-disubstituted benzenes offer only a single accessible C–H bond (Scheme 6). Yet, the observed rate increase from *m*-xylene to *o*-xylene is approximately 5-fold. Further, the reaction of 1,3-dimethoxybenzene produces <1 TO, while 1,2-dimethoxybenzene is the fastest 1,2-disubstituted benzene tested. An approximate 2-fold increase in rate is seen for both 1,3-dichlorobenzene compared to 1,2-dichlorobenzene and 1,3-bis­(trifluoromethyl)­benzenes compared to 1,2-bis­(trifluoromethyl)­benzene.

**Scheme 6:**
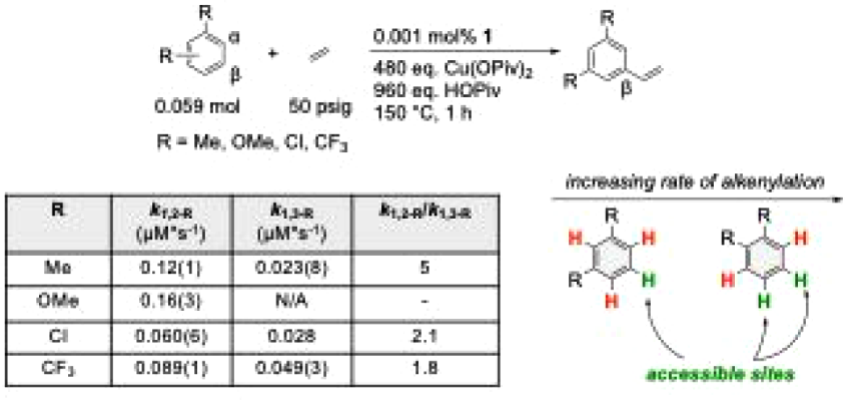
Ratio of Rates for Ethenylation of 1,2-disubstitued Benzenes Compared to 1,3-disubstituted Benzenes^a^

a Number of accessible C–H bonds for 1,3- and 1,2-disubstituted benzenes explaining the steric effect on the rate of alkenylation. 1,2-R = 1,2-disubstituted benzene and 1,3-R = 1,3-disubstituted benzene

As for the electronic effects, the rate of ethenylation for 1,3-disubstituted benzenes is higher for electron-poor benzenes, with *m*-CF_3_ being the fastest substrate and *m*-OMe being the slowest substrate (Scheme 7). In contrast, the rate of ethenylation for 1,2-disubstituted benzenes is higher for electron-rich benzenes, with *o*-OMe being the fastest substrate and *o*-Cl being the slowest substrate. This trend is the opposite of the observed trend for 1,3-disubstituted benzenes; that is, electron-donating substituents enhance the rate of ethenylation for 1,2-disubstituted benzenes. Although the rates of ethenylation between 1,2- and 1,3-disubstituted benzenes show opposing trends, these data do not explain the unusually slow reaction rate of the *m*-OMe substrate. For this reason, we believe that there is a combination of substituent electronics (i.e., inductive and resonance effects) and reaction site (i.e., the selectivity of alkenylation) that will affect the observed relative rates for these substrates.

**Scheme 7:**
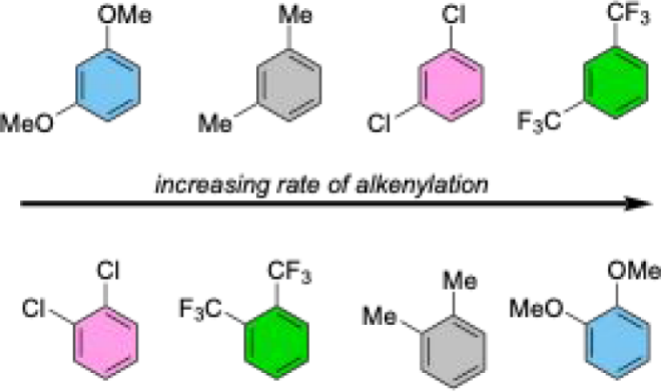
Observed Trend for Rates of Alkenylation for 1,3- and 1,2-disubstituted Benzenes Showing an Electronic Effect

To further investigate the electronic effects that the substituents have on the rate of arene ethenylation, we explored Hammett correlation plots (Figure 3). The *meta-*Hammett parameter (σ_m_) was used for the 1,3-disubstituted benzenes, and the *para-*Hammett parameter (σ_p_) was used for the 1,2-disubstituted benzenes. The rates of reaction were determined using the initial time point (i.e., method of initial rates) to avoid any complications from partial catalyst decomposition and concentration change. Rates of 1,2-disubstituted benzenes were adjusted to control for the number of possible products being twice that of the 1,3-disubstituted benzenes, considering the β-selectivity. The Hammett plot shows a deviation from linearity for the *o-*Me and *o*-OMe points, suggesting that these substrates could be reacting by a different mechanism compared with the other substrates tested.

**Figure 3:**
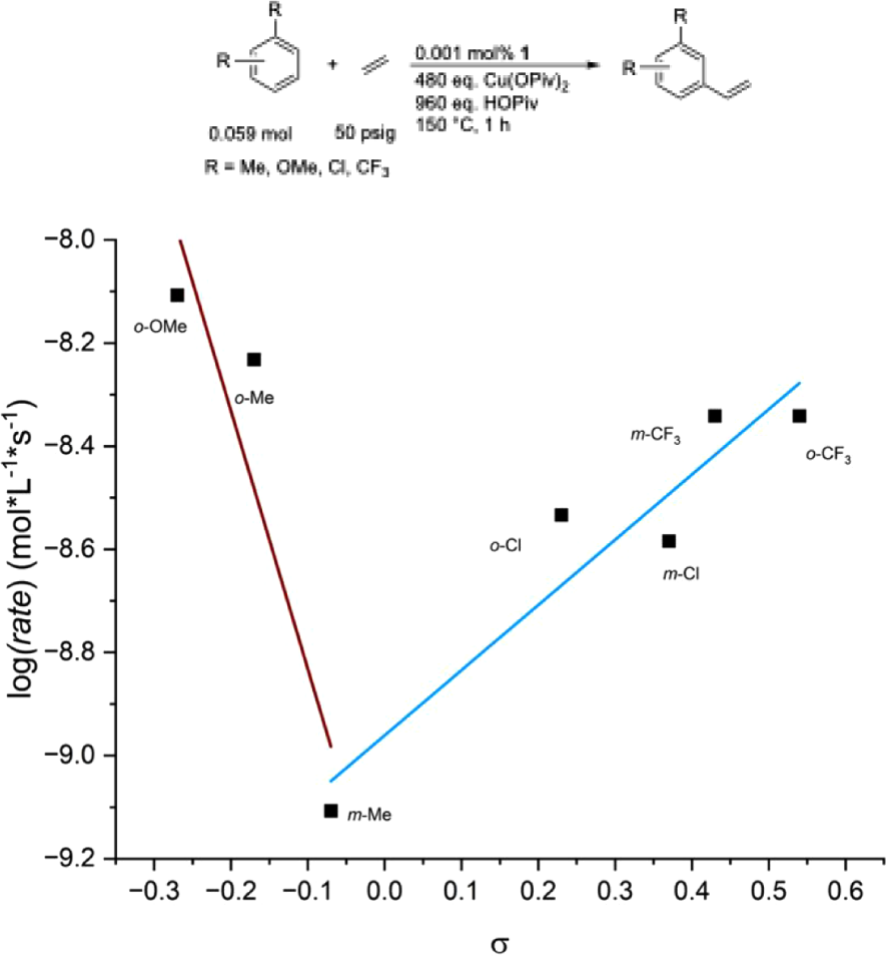
Hammett correlation plot for alkenylation of 1,2- and 1,3-disubstituted benzenes. Reaction conditions: 0.059 mol of arene, 0.001 mol % [(η^2^-C_2_H_4_)_2_Rh­(μ-OAc)]_2_ (relative to arene), 480 eq. Cu­(OPiv)_2_ (relative to a single Rh atom), 960 eq. HOPiv (relative to a single Rh atom), 50 psig ethylene, 150 °C. Quantified using GC-FID analysis with HMB used as an internal standard.

Previously, we have reported that heating [(η^2^-C_2_H_4_)_2_Rh­(μ-OAc)]_2_ in the presence of Cu­(OPiv)_2_ will form the heterobimetallic species [(η^2^-C_2_H_4_)_2_Rh­(μ-OPiv)_2_]_2_(μ-Cu), which we proposed as an active catalyst for the production of styrene from benzene and ethylene (Scheme 8).^48^ DFT calculations revealed that there is a rate benefit to the incorporation of Cu­(II) into the catalyst structure compared to mono-Rh and bis-Rh structures without Cu­(II). The predicted activation barrier for styrene production starting from [(η^2^-C_2_H_4_)_2_Rh­(μ-OAc)]_2_ is too high compared to the experimental kinetics; however, starting from [(η^2^-C_2_H_4_)_2_Rh­(μ-OPiv)_2_]_2_(μ-Cu), the experimental kinetics are in agreement with the calculated activation barriers for the catalytic steps. Furthermore, our DFT calculations revealed that the lowest energy pathway for benzene C–H activation is a stepwise oxidative addition and reductive coupling step (Scheme 7, see pathway II), but a concerted-metalation deprotonation (CMD) pathway was calculated to be only 1 kcal/mol higher in energy, indicating competition (Scheme 7, see pathway I).^48^ Thus, based on the calculations, for benzene functionalization, there is only a slight energy difference between the CMD and the oxidative addition/reductive coupling pathway. Based on experimental results from the Hammett plot, we hypothesized that the mechanism of C–H activation is different for electron-rich and electron-poor arenes. From our previous DFT calculations indicating that benzene C–H activation has a small energy difference between the two C–H activation pathways, it is possible that for electron-rich arenes, one C–H activation mechanism has a lower energy than the other. This suggests that the reverse would be true for electron-poor arenes; that is, one C–H activation mechanism is lower in energy than the other mechanism.

**Scheme 8:**
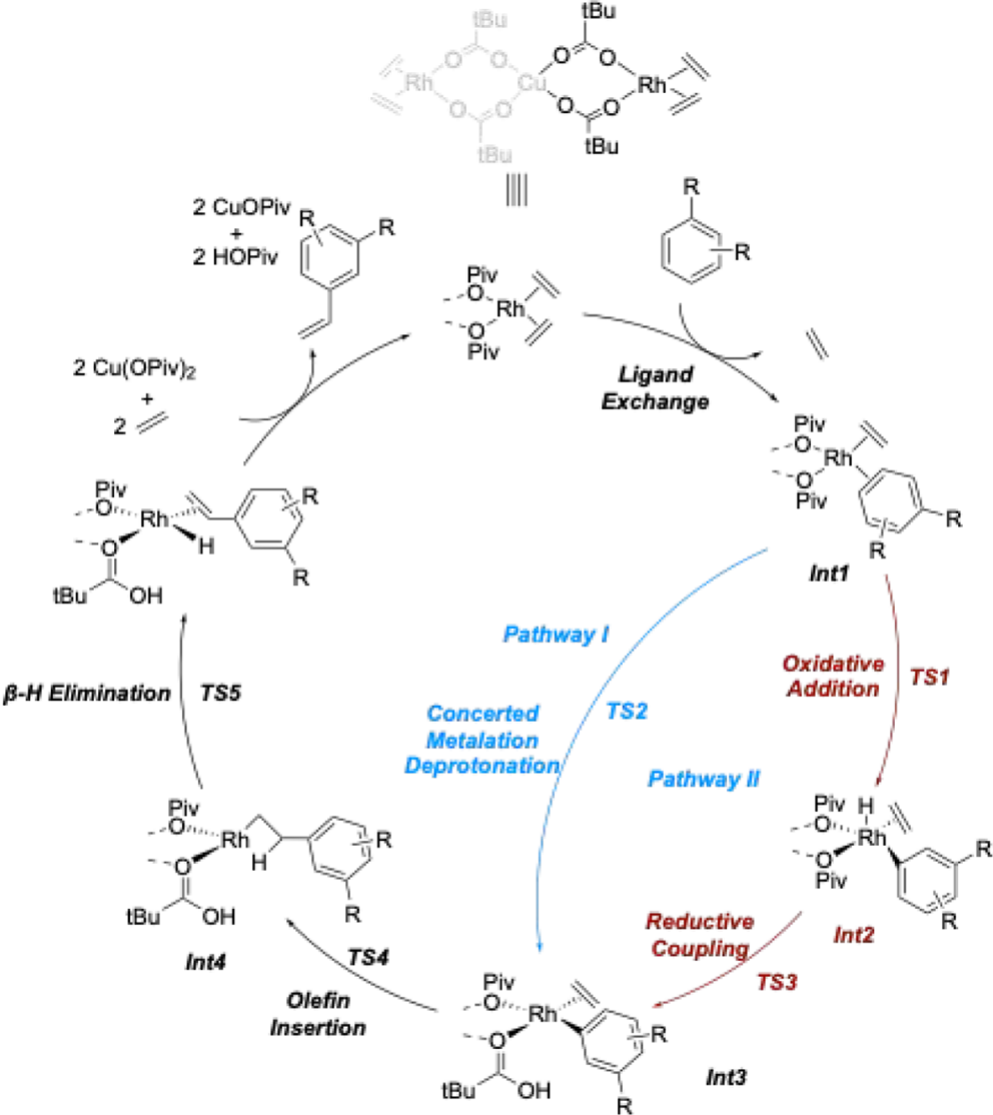
Catalytic Cycle of Rh-catalyzed Disubstituted Arene Ethenylation^a^

a Shown in red and blue are the two proposed C–H activation steps that are the focus of this study

### QM-Based Computational Investigations

To understand our experimental observations, we sought to probe the mechanism of C–H bond activation as a function of arene substituent identity, with a focus on oxidative addition/reductive coupling versus CMD. We performed QM-based computational investigations at the (U)­B3LYP-D3/def2-SVP-CPCM­(benzene)//(U)­B3LYP-D3/def2-TZVP-CPCM­(benzene) level of theory. Taking previous investigations as a reference,^48^ we considered it reasonable to simplify pivalate (OPiv) as acetate (OAc). For the implicit solvent model, the reaction substrate could be used, but the necessary physical parameters (e.g., dielectric constant and refractive index) are not consistently available or measured under comparable conditions for all substrates. Thus, to avoid introducing additional uncertainties, we used benzene as the implicit solvent for all substrates to capture relative energy relationships and reveal the underlying trends.

We set [(η^2^-C_2_H_4_)_2_Rh­(μ-OAc)]_2_ and [Cu­(OPiv)_2_]_3_ trimer as our reference state of 0.0 kcal/mol since they are the most stable forms of the catalyst and Cu­(II) precursor.^48^ Arenes coordinate to Rh by displacing an ethylene ligand, resulting in an energy increase ranging from 2.1 to 6.2 kcal/mol (e.g., Figures 4 and 5). Electron-rich arenes are preferred, likely due to their stronger σ-donating ability, as η^2^-coordinated ligands. In the next step, two pathways for arene C–H activation are possible (oxidative addition or CMD). Key results from our computational studies are given below.

**Figure 4:**
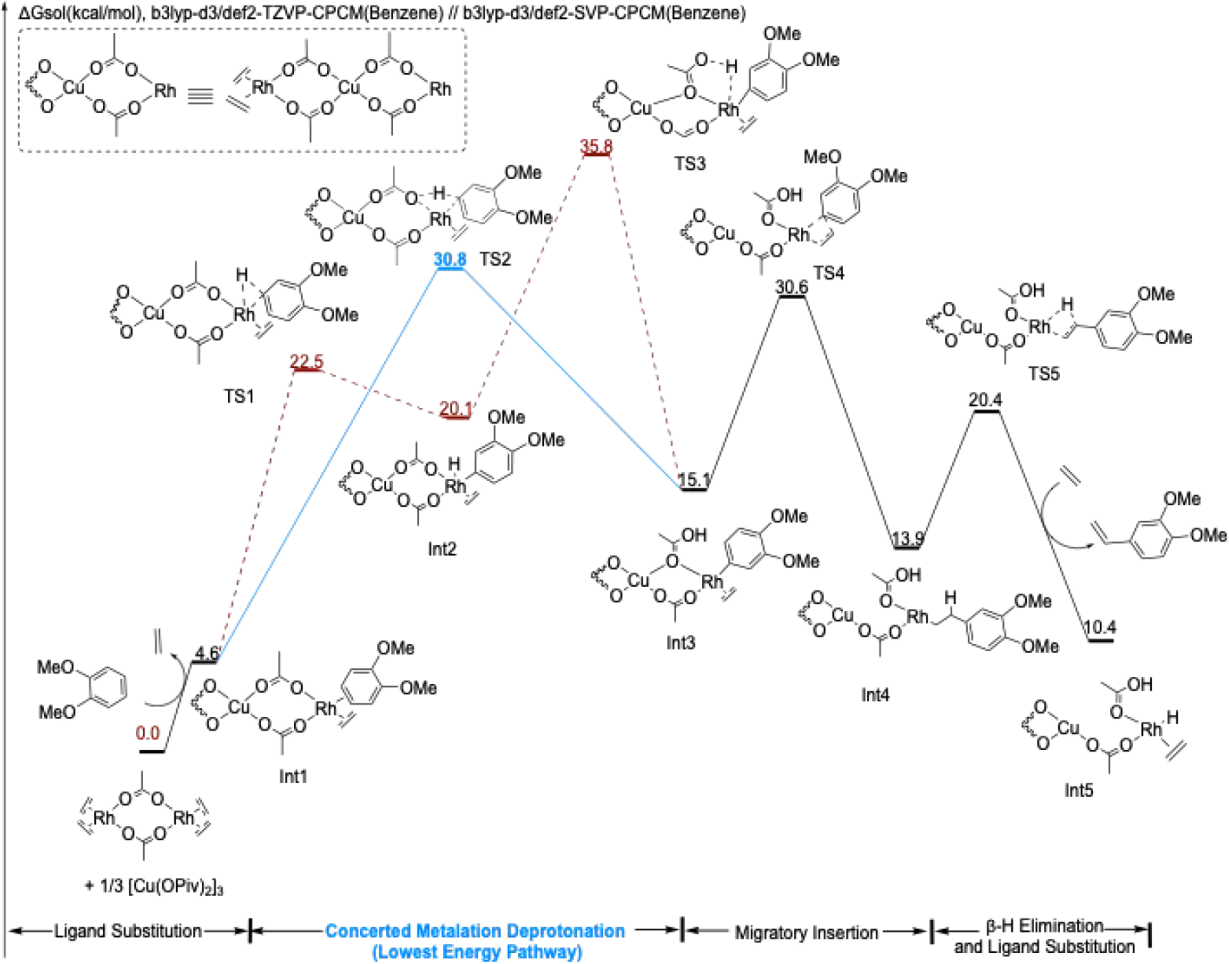
DFT-computed free energy changes for vinyl arene formation with 1,2-dimethoxybenzene. Computations were performed at the (U)­B3LYP-D3/def2-SVP-CPCM­(benzene)//(U)­B3LYP-D3/def2-TZVP-CPCM­(benzene) level of theory. Energies are given in kcal/mol. Relative energies are not to scale. The **red pathway** shows the energetics of the reaction pathway with C–H activation by oxidative addition/reductive coupling (TS1 and TS3). The **blue pathway** shows the energetics of the reaction pathway with C–H activation by concerted-metalation deprotonation (TS2). The **solid line** indicates the lowest energy pathway for the 1,2-dimethoxybenzene substrate, while the **dashed line** indicates the higher energy pathway.

**Figure 5:**
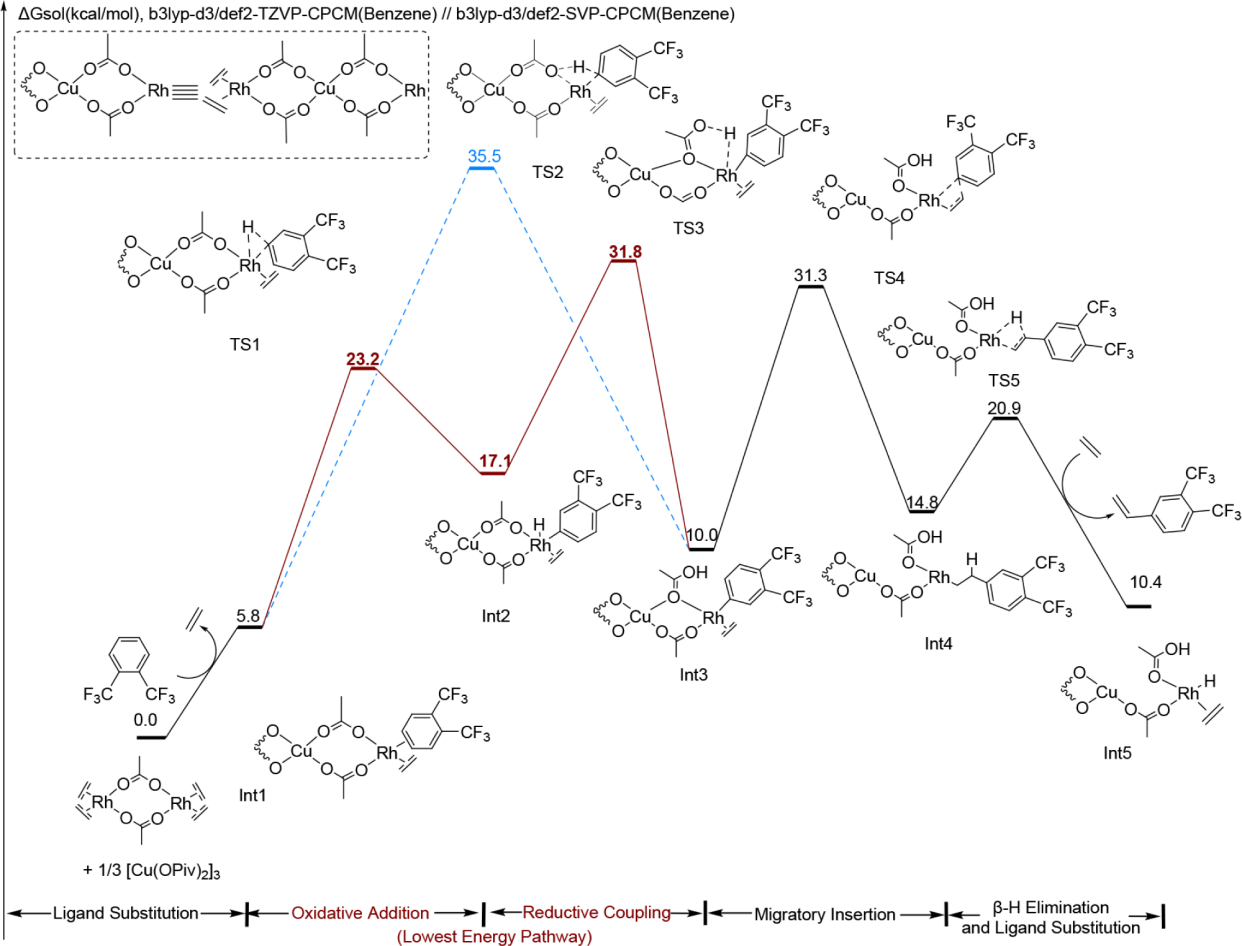
DFT-computed free energy changes of substituted styrene formation for *o*-CF_3_. Computations were carried out at the (U)­B3LYP-D3/def2-TZVP-CPCM­(benzene)//(U)­B3LYP-D3/def2-SVP-CPCM­(benzene) level of theory. Energies are given in kcal/mol. Relative energies are not to scale. The **red pathway** shows the energetics of the reaction pathway with C–H activation by oxidative addition/reductive coupling (TS1 and TS3). The **blue pathway** shows the energetics of the reaction pathway with C–H activation by concerted-metalation deprotonation (TS2). The **solid line** indicates the lowest energy pathway for the 1,2-bis­(trifluoromethyl)­benzene substrate, while the **dashed line** indicates the higher energy pathway.

Figure 4 shows the entire process for the conversion of *o*-OMe to vinyl arene. Either pathway for C–H activation, oxidative addition or CMD, ultimately leads to Int3, from which ethylene insertion into the Rh-aryl bond is followed by β-hydride elimination. From Int3 in Figure 4, the activation barrier for ethylene insertion (TS4) is calculated to be 15.5 and 30.6 kcal/mol above the reference state.

Figure 5 shows the calculated overall reaction pathway for the conversion of *o*-CF_3_ to vinyl arene. From Int3 in Figure 5, the activation barrier for ethylene insertion (TS4) is calculated to be 21.3 kcal/mol, which is 31.3 kcal/mol above the reference state. It is interesting to note that the energy for the conversion of Int3 to TS4 is much higher for the *o*-CF_3_ (21.3 kcal/mol) than for the *o*-OMe substrate (15.5 kcal/mol), but the activation energy from the reference state to TS4 is similar (31.3 kcal/mol for *o*-CF_3_ versus 30.6 kcal/mol for *o*-OMe). Thus, it is the relative stability of Int3 as a function of aryl identity that is the primary determinant for the activation energy for ethylene insertion.

For six disubstituted arene substrates, Figure 6 shows the calculated energy barriers for C–H bond activation by oxidative addition/reductive coupling (blue circles) and CMD (red squares). The *o*-OMe substrate exhibits a lower energy barrier via a CMD transition state (Figure 6 TS2, 30.8 kcal/mol) than the two-step oxidative addition/reductive coupling pathway (Figure 6 TS3, 35.8 kcal/mol). Likewise, our calculations show that *m*-Me and *o*-Me have overall lower energy barriers for the CMD pathway (Figure 6 TS2, 33.0 and 31.4 kcal/mol, respectively) compared to oxidative addition and reductive coupling (Figure 6 TS3, 35.2 and 35.8 kcal/mol, respectively).

**Figure 6:**
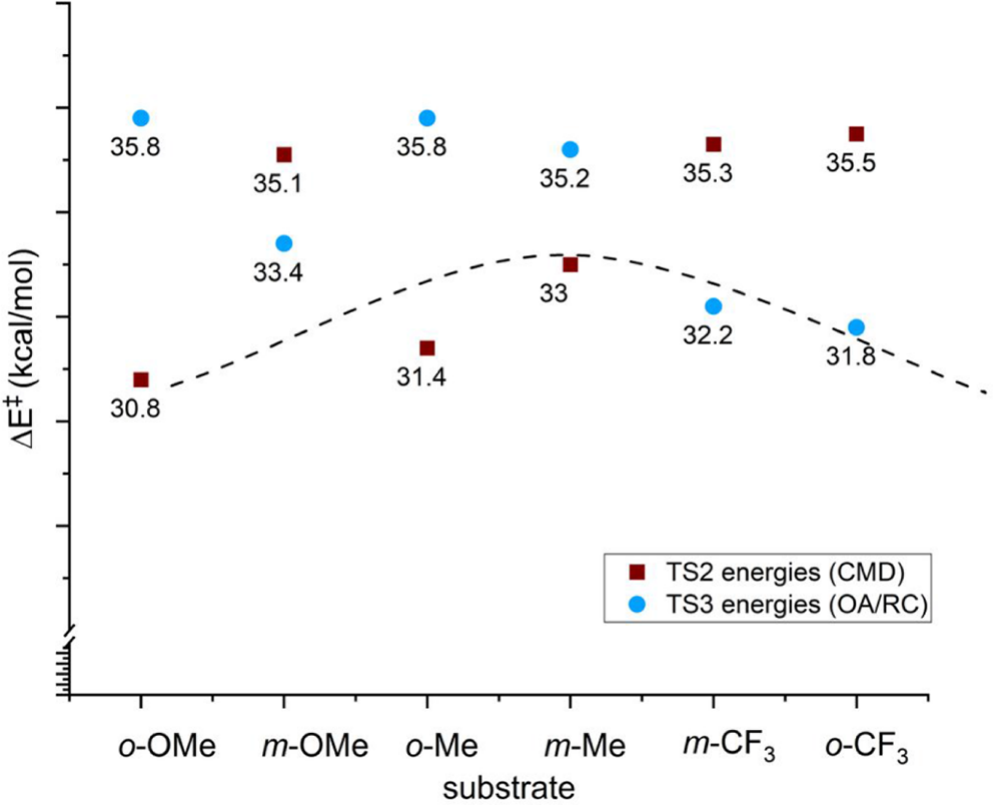
Energies for C–H activation by concerted-metalation deprotonation (CMD) in red squares (TS2 in Figures 4 and 5) and oxidative addition/reductive coupling (OA/RC) in blue circles (the higher energy of TS1 or TS3 in Figures 4 and 5) for six substrates. Note that for the oxidative addition/reductive coupling pathway, TS3 is calculated, in all cases, to be higher in energy than TS1. Thus, the energies of TS3 are shown above. Energies are given in kcal/mol. Relative energies are not to scale. The dotted line is intended to guide the eye to the lowest TS energy for each substrate.

In contrast to the *o*-OMe, *o*-Me, and *m*-Me substrates, the *o*-CF_3_, *m*-CF_3_, and *m*-OMe substrates undergo oxidative additions and reductive couplings with lower overall activation barriers (Figure 6, TS3, 31.8, 32.2, and 33.4 kcal/mol, respectively) compared to the CMD pathway (Figure 6 TS2, 35.5, 35.3, and 35.1 kcal/mol, respectively). It should be noted that for the substrate *m*-OMe, the barriers for both C–H activation mechanisms are relatively high (Figure 6, TS2 = 35.1 kcal/mol and TS3 = 33.4 kcal/mol), which is consistent with our experimental results that show <1 TO of product after 1 h of reaction.

For the six substrates calculated here, our results indicate that C–H activation is likely the rate-determining step, although in some cases the activation barrier for olefin insertion is similar in energy to the C–H bond-breaking step. For the three ortho-disubstituted substrates calculated, the relative activation barriers for C–H activation predict the following relative rates: *o*-OMe (30.8 kcal/mol) < o-Me (31.4 kcal/mol) < *o*-CF_3_ (31.8 kcal/mol), which is consistent with experimental comparisons for the rate of ethenylation (Figure 1). For the three meta-disubstituted substrates calculated, the relative activation barriers for C–H activation predict the following relative rates: *m*-CF_3_ (32.2 kcal/mol) < *m*-Me (33.0 kcal/mol) < *m*-OMe (33.4 kcal/mol), which is also consistent with the experimental comparisons of the relative rates of ethenylation (Figure 2).

Our calculations are consistent with experimental observations and indicate that the reaction mechanism for C–H activation likely switches between two competing pathways: (1) the direct TS2 route (i.e., CMD) and (2) the TS1 and TS3 (i.e., C–H oxidative addition and O–H reductive coupling) sequence, with the competition centered on their respective rate-limiting steps, TS2 and TS3 (Figure 7). For all six substrates calculated, C–H bond breaking by oxidative addition (TS1) has a lower activation barrier than CMD (TS2), but it has a high energy for O–H reductive coupling of the −H, which renders the oxidative addition pathway less favorable than the CMD pathway for some substrates. As a reductive coupling transition state, the energy of TS3 is possibly primarily governed by the acidity of the Rh–H bond in intermediate Int3, which is, in turn, modulated by the electronic properties of the arene ligands. Arenes bearing electron-withdrawing groups (EWGs), such as *m*-CF_3_ and *o*-CF_3_, reduce the electron density at the Rh center, thereby rendering the Rh–H bond more acidic and stabilizing TS3. This provides an explanation for why the preferred mechanism for arene C–H functionalization changes to the oxidative addition/reductive coupling pathway for the CF_3_ substituted arenes.

**Figure 7:**
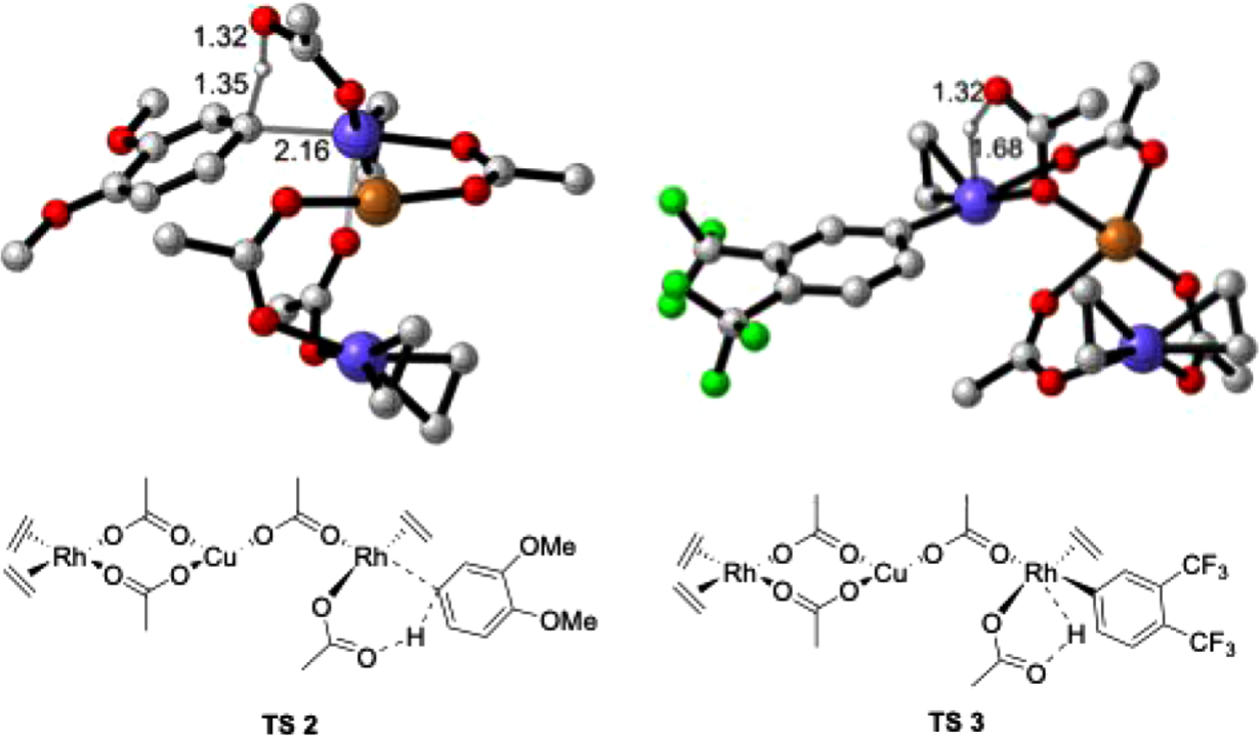
DFT-optimized representative structures: CMD transition state (TS2) for 1,2-dimethoxybenzene (left) and reductive coupling transition state (TS3) for 1,2-bis­(trifluoromethyl)­benzene (right). Nonessential hydrogen atoms are omitted for clarity. (Gray, C; white, H; red, O; green, F; purple, Rh; copper-brown, Cu).

To elucidate the key factors governing the CMD process, we performed distortion-interaction-activation strain (DIAS) analyses (Figure 8). By decomposing the activation energy into the distortion energies of the substrate (*E*_dist_ Ar–H) and catalyst (*E*_dist_ Rh), as well as the interaction energy (*E*_int_) between these two components, we obtain a clearer understanding of the factors responsible for the energy increase in the transition state (TS2) relative to that of the pre-TS intermediate. Along the series from EDG-substituted arenes to EWG-substituted arenes, we observed two opposing trends. The distortion energy of the arene substrates decreases as the negative charge accumulated on the reactive carbon (Cα) is better stabilized by EWGs. That is, the more electron-poor arene substrates have a decreased arene distortion energy (Figure 8, *E*_dist_ Ar–H). Conversely, the interaction energy decreases as the decreased electron density weakens the interaction. That is, the more electron-poor substrates have a decreased interaction energy (Figure 8, *E*_int_). While these effects partially offset each other, the interaction energy plays a more dominant role overall. Consequently, the more electron-rich substrates have the highest interaction energy and therefore stabilize the CMD transition state (TS2).

**Figure 8:**
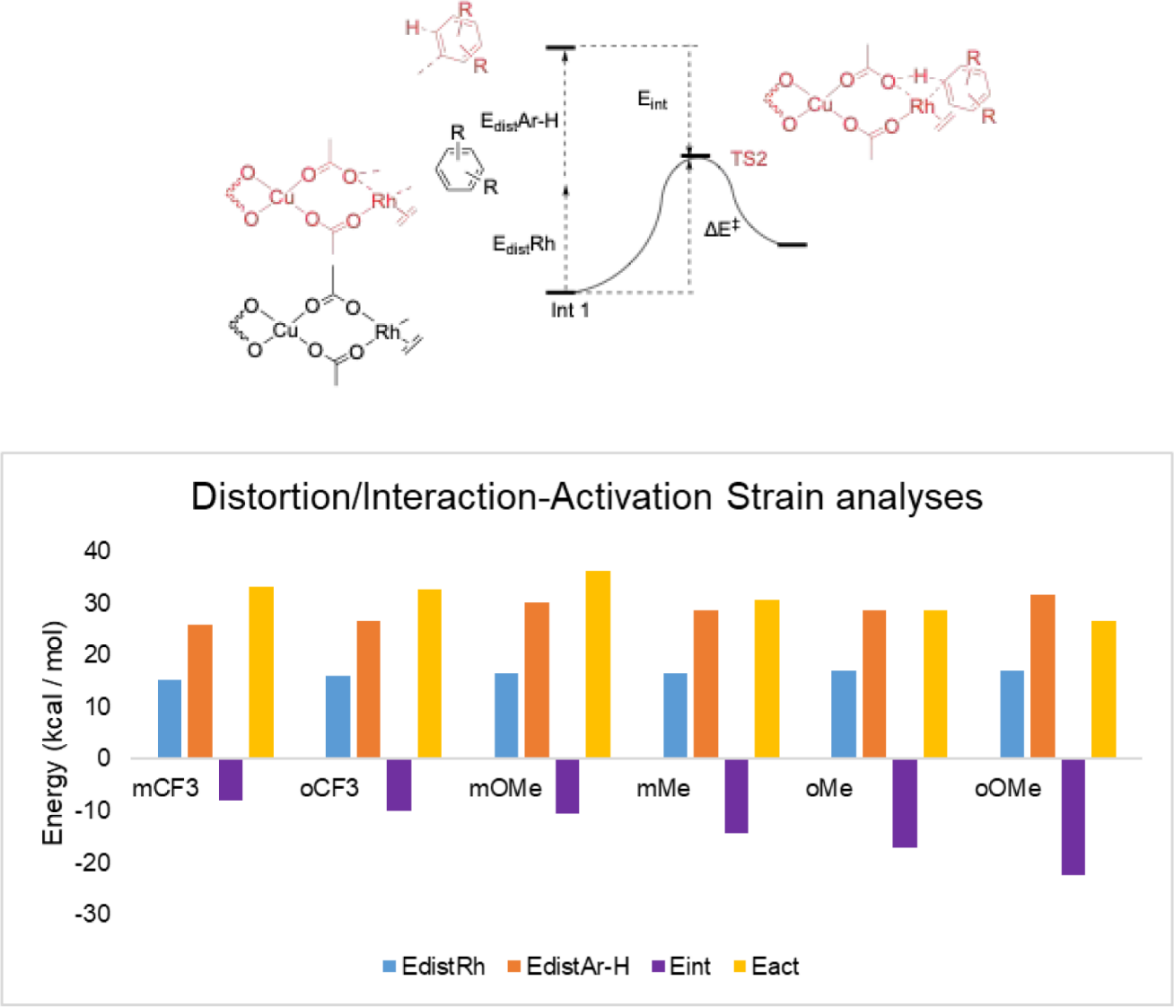
Distortion/interaction-activation strain (DIAS) analysis of the concerted metalation-deprotonation (CMD) transition states reveals the key factors for TS2. Free energies were calculated at the (U)­B3LYP-D3/def2-SVP-CPCM­(benzene)//(U)­B3LYP-D3/def2-TZVP-CPCM­(benzene) level of theory. Fragment distortion and interaction energies were calculated at the (U)­B3LYP-D3 level of theory with the def2-TZVP basis set without the inclusion of solvation energy corrections (blue, distortion energies of Rh catalyst; orange, distortion energies of arenes; purple, interaction energies; yellow, activation energies). Energies are given in kcal/mol.

Our results highlight *m*-OMe as an outlier. We observe experimentally very slow catalytic ethenylation with this substrate. Also, consistent with the experimental results, our calculations predict that *m*-OMe has the highest activation barrier for C–H activation (Figure 5). To better understand this observation, we should consider both the overall electronic density and the position of the reaction site. As illustrated in Scheme 9, the methoxy group has a strong negative inductive effect (decreasing the electron density, transmitting along σ-bonds, and diminishing rapidly with increasing distance) and a strong conjugative effect (propagating through conjugated systems, where influence alternates along the π-framework). Consequently, the 5-position of *m*- is mainly influenced by the negative inductive effect. To substantiate this point, we calculated natural population analysis (NPA) charges for TS2 with all six substrates. Based on the theoretical analysis, we can qualitatively derive the influence of substituents on the carbon bond to Rh (Table 3, left), which aligns with computational results (Table 3, right). In Table 3, we see that the reactive carbon of *m*-OMe has a relatively low negative charge, explaining the low interaction energy with the catalyst part shown in DIAS analysis (Figure 8). Therefore, *m*-OMe does not prefer the CMD transition state (TS2) that is preferred by the other electron-rich substrates.

**Table 3:** Natural Population Analysis (NPA) Charges in TS2 with Different Substrates

Q_NPA_	Theoretical qualitative derivation (Cα)	Computational analysis (Cα)
*m*-CF3	Less positive	–0.17
*o*-CF3	Positive	–0.16
*m*-OMe	Positive	–0.16
*m*-Me	Negative	–0.18
*o*-Me	More negative	–0.19
*o*-OMe	More negative	–0.19

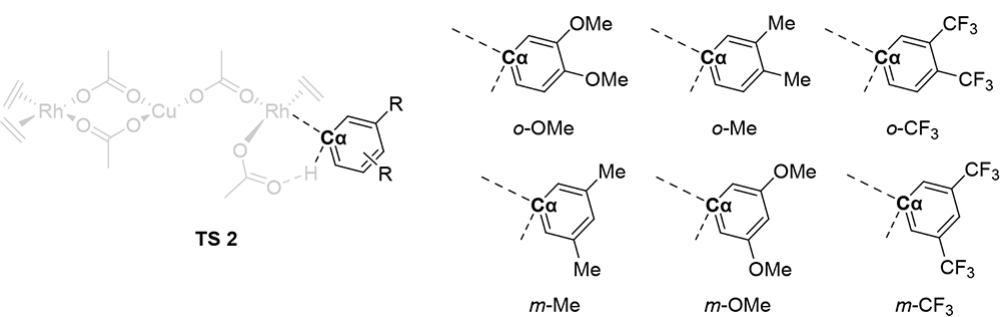

**Scheme 9:**
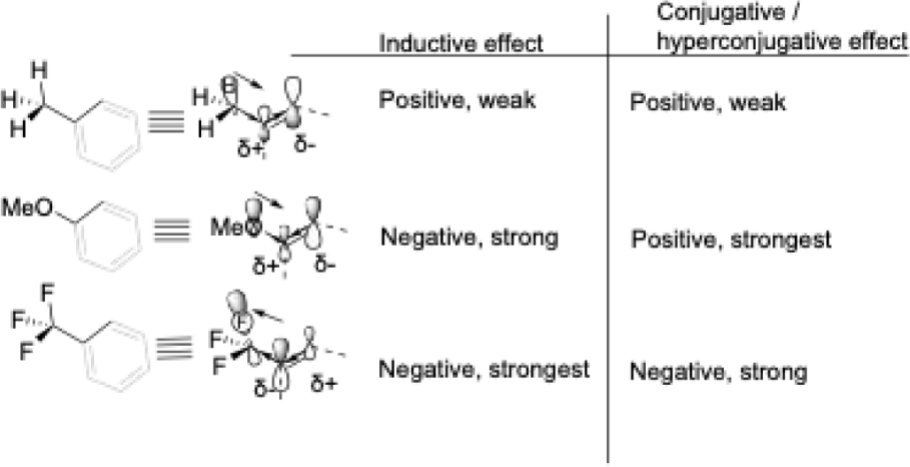
Conjugative and Inductive Effect of Selected Substituents^a^

a Arrows along the σ-bonds indicate the inductive effect. Arrows above the bonds indicate the conjugative effect. The direction that the arrow points represents how electrons are influenced

## Summary and Conclusions

We have demonstrated the catalytic oxidative ethenylation of 1,3- and 1,2-disubstituted benzenes using the catalyst precursor [(η^2^-C_2_H_4_)_2_Rh­(μ-OAc)]_2_ and Cu­(OPiv)_2_ as the *in situ* oxidant. The rate of ethenylation is influenced by both steric and electronic factors of the arene substituents. Ethenylation reactions of 1,2-disubstituted benzenes are faster compared to 1,3-disubstituted benzenes, regardless of the arene substituent, which we propose arises primarily because the number of accessible C–H bonds (i.e., C–H bonds not directly adjacent to a substituent) is greater for 1,2-disubstituted benzenes relative to 1,3-disubstituted benzenes. For 1,2-disubstituted benzenes, the rate of ethenylation increases with more electron-donating substituents (i.e., *o*-OMe > *o*-Me > *o*-CF_3_ > *o*-Cl). For 1,3-disubstituted benzenes, the rate of ethenylation generally increases with more electron-withdrawing substituents (i.e., *m*-CF_3_ > *m*-Cl > *m*-Me > *m*-OMe).

Using DFT calculations, we showed that the mechanism of C–H activation can shift between two pathways: (1) a concerted metalation-deprotonation transition state (TS2) and (2) an oxidative addition transition state (TS1) followed by reductive coupling (TS3). Electron-withdrawing group-substituted arenes stabilize the negative charge on Rh, thereby likely lowering the energy of TS3, which involves a formal proton transfer to an acetate ligand. In contrast, electron-donating group (EDG)-substituted arenes that enhance the bonding interaction in TS2 significantly reduce the activation barrier. The *m*-OMe substrate has been identified as an outlier both experimentally (i.e., unusually slow relative rate of ethenylation) and computationally (i.e., high calculated energy barriers for both TS2 and TS3). The poor reactivity of the *m*-OMe substrate is proposed to be due to the relative positions of the substituents and the reactive site. The methoxy groups (situated three bonds away from the reactive carbon) do not significantly donate electron density through conjugation to the reactive carbon, which would stabilize the CMD transition state (TS2), nor do they withdraw electron density through inductive effects, which would stabilize reductive coupling transition states (TS3).

Further advancements in the field of C–H functionalization are needed to increase the scope of accessible reactions to provide practical tools in organic synthesis. The discovery of new C–H functionalization reagents is important, and a fundamental understanding of C–H activation mechanisms can drive the development of new catalysts that will ultimately enhance the field of C–H functionalization. We propose that for a set of relatively “simple” arene substrates, the same catalyst undergoes two different C–H activation mechanisms. Each arene substrate has unique steric and electronic properties that will ultimately modify the interaction with the catalyst fragment and thus favor one C–H activation pathway over the other. The observation that substrates that are similar in structure can give rise to measurable differences in the C–H activation pathway demonstrates a fine line between accessible mechanisms for this specific reaction and the catalyst.

## Experimental Section

### General Considerations

Unless otherwise noted, all modifications were carried out under an inert atmosphere in a dinitrogen-filled glovebox. Glovebox purity was maintained by periodic N_2_ purges to maintain a dioxygen concentration of <20 ppm. Ethylene and propylene (99.9%) were purchased in gas cylinders from a commercial source and used as received. All liquid reagents were degassed for 30 min using N_2_ before being stored inside a glovebox prior to use. *o*-xylene (99%) was purchased from Fisher Scientific. *m*-xylene (99%) was purchased from a commercial source. 1,2-Dimethoxybenzene (98%) was purchased from a commercial source. 1,3-dimethoxybenzene (98%) was purchased from a commercial source. 1,2-dichlorobenzene (98%) was purchased from TCI. 1,3-dichlorobenzene (99%) was purchased from Oakwood. α,α,α,α’,α’,α’-hexafluoro-*o*-xylene (>97%) was purchased from TCI America. α,α,α,α’,α’,α’-hexafluoro-*m*-xylene (98+%) was purchased from Ambeed. 2-chlorotoluene (99%) was purchased from Apollo Scientific. 2-chloro-α,α,α-trifluorotoluene (99%) was purchased from Apollo Scientific. 2-chloroanisole (98%) was purchased from Thermo Scientific Chemicals. 2-methylanisole (99%) was purchased from a commercial source. 1-methyl-2-(trifluoromethyl)­benzene (98%) was purchased from a commercial source. Di-μ-acetatotetrakis­(η^2^-ethene)­dirhodium­(I) (**1**) was synthesized according to a previously published procedure.^56^ Copper­(II) pivalate was synthesized according to a previously published procedure.^57^ Reactions were performed in glass Fisher-Porter reactors equipped with a pressure gauge and a proportional relief valve. All reactions were performed behind a polycarbonate blast shield (4.7 mm thickness and 30″ height) to protect the operator. Gas chromatography/mass spectrometry (GC-MS) was performed using a Shimadzu GCMS-QP2020 NX instrument with a 30 × 0.25 mm Rxi-5 ms column with a 0.25 μm film thickness using electron impact ionization. Gas chromatography-flame ionization detector (GC-FID) was performed using a Shimadzu GC-2014 instrument with a 30 × 0.32 mm DB-5 ms UI column with a 0.25 μm film thickness. For the GC-FID instrument, TOs were quantified by linear regression analysis of chromatograms using the authentic product or estimated using compounds of similar molecular weights and composition. Plots of peak area versus molar ratio gave regression lines relative to those of the internal standard hexamethylbenzene. Slopes and correlation coefficients for the following compounds are as follows: 3,4-dimethlstyrene (0.67, 0.9998), 3,5-dimethoxystyrene (3.01, 0.9989), 3,4-dichlorostyrene (0.38, 0.9986), and 3,5-bis­(trifluoromethyl)­styrene (0.85, 0.9993).

### Representative Procedure for Disubstituted Benzene Alkenylation

A 10 mL stock solution of [(η^2^-C_2_H_4_)_2_Rh­(μ-OAc)]_2_ (340 μg, 1.55 μmol) was prepared in arene. To oven-dried four-dram vials, 1 mL of stock solution, Cu­(OPiv)_2_ (74 mg, 279 μmol, 480 eq. per Rh atom), HOPiv (57 mg, 558 μmol, 960 eq. per Rh atom), HMB (2.0 mg, 15.1 μmol, 20 eq. per Rh atom), and the appropriate amount of arene were added. The contents of the 4-dram vials were transferred into the previously described glass Fisher-Porter reactors, sealed, and removed from the glovebox. Ethylene (50 psig) was added to each reactor by using a high-pressure gas manifold. Reactors were stirred and heated at 150 °C using an oil bath on a hot plate. After it was cooled to room temperature, an aliquot of this solution was diluted with DCM and washed with a saturated solution of NaHCO_3_ prior to GC-FID or GC-MS analysis.

### Computational Details

All density functional theory (DFT) calculations were conducted with the Gaussian 16 software package.^58^ Geometry optimizations of all the intermediates and transition states were performed at the B3LYP^59,60^ level of theory with the def2-SVP^60^ basis set, including Grimme’s D3 dispersion corrections.^61^ Vibrational frequency analyses were conducted at the same level of theory to evaluate its zero-point vibrational energy (ZPVE) and thermal corrections at 298 K. Single-point energies were computed at the same level of theory using the def2-TZVP^62^ basis set, including solvation energy corrections. The solvation energies were evaluated by a self-consistent reaction field (SCRF) using the CPCM model.^63^ Fragment distortion and interaction^64–69^ energies were calculated at the B3LYP^60,64^ level of theory with the def2-TZVP^60,62^ basis set, including Grimme’s D3 dispersion corrections^61^ in the gas phase. Extensive conformational searches for the intermediates and transition states were conducted to ensure that the lowest-energy conformers were located. Intrinsic reaction coordinate (IRC) calculations of the transition states were performed to verify their locations along the free energy surface. The three-dimensional diagrams of the molecules were generated using CYLView,^70^ with hydrogen atoms omitted for clarity. The natural population analyses were calculated using the NBO^71,72^ module of Gaussian 16 and Multiwfn.^73,74^

## Supplementary Material





## Notes

GC-FID chromatograms, additional experimental details, materials, photographs of experimental setup, additional computational results, and energies of DFT-computed stationary points (PDF)
An xyz file containing coordinates (XYZ)
