# Supplementary material for: A Change in C–H Activation Mechanism: Experimental and Computational Investigations of Rh-Catalyzed Disubstituted Benzene Functionalization
Source: Organometallics. 2025 Oct 29;44(21):2579–91. doi: 10.1021/acs.organomet.5c00379 (PMC12606707; doi:10.1021/acs.organomet.5c00379)
Supplement: Supplementary file 1 [file om5c00379_si_001.pdf]

Supporting Information for

A change in C–H activation mechanism: experimental and computational investigations of Rh-catalyzed disubstituted benzene functionalization

Christopher W. Reid[†ϕ], Chi Zhang[‡ϕ], Lauren E. Baptiste[†], K. N. Houk,[#]

William A. Goddard III[‡*] and T. Brent Gunnoe[†*]

[†] Department of Chemistry, University of Virginia, Charlottesville, Virginia 22904, United States

[‡] Materials and Process Simulation Center, California Institute of Technology, Pasadena, California 91125, United States

[#]Department of Chemistry and Biochemistry, University of California, Los Angeles, California 90095-1569, United States

[ϕ] Authors contributed equally.

* Correspondence to: tbg7h@virginia.edu, wag@caltech.edu

Table of Contents

Figure S1. Photographs of: A) 3 oz Fisher-Porter reactor with reactor top fitted with adjustable pressure poppet check valve and pressure gauge, B) stainless steel reactor fitted with adjustable pressure poppet check valve and pressure gauge, C) two oil baths behind a blast shield, D) two heated aluminum blocks behind blast shield, and E) close-up of adjustable pressure poppet check valve. 3

Figure S2. Representative GC-FID chromatogram for o-xylene ethenylation. 4

Figure S3. Representative GC-FID chromatogram for *m*-xylene ethenylation. 4

Figure S4. Representative GC-FID chromatogram for 1,2-dimethoxybenzene ethenylation. 5

Figure S5. Representative GC-FID chromatogram for 1,3-dimethoxybenzene ethenylation. 5

Figure S6. Representative GC-FID chromatogram for 1,2-dichlorobenzene ethenylation. 6

Figure S7. Representative GC-FID chromatogram for 1,3-dichlorobenzene ethenylation. 6

Figure S8. Representative GC-FID chromatogram for 1,2-bis(trifluoromethyl)benzene ethenylation. 7

Figure S9. Representative GC-FID chromatogram for 1,3-bis(trifluoromethyl)benzene ethenylation. 7

Figure S10. Energy profiles for all six substrates. DFT-computed free energy changes of substituted styrene formation. Computations are carried out at (U)B3LYP-D3/def2-SVP-CPCM(Benzene) // (U)B3LYP-D3/def2-TZVP-CPCM(Benzene) level of theory. Energies are given in kcal/mol. Mechanisms for different substrates are differentiated by colors: red, 1,3-bis(trifluoromethyl)benzene; orange, 1,2-bis(trifluoromethyl)benzene; dark blue, m-xylene; light blue, o-xylene; dark purple, 1,2-dimethoxybenzene; light purple, 1,3-dimethoxybenzene. 8

Table S1. Energies in Figure S10 and thermal correction to Gibbs Free Energy (TCG) was calculated with geometry optimization at the b3lyp-d3/def2-svp-CPCM (C_6H_6) level of theory, and single point energy (E) was calculated at the b3lyp-d3/def2-tzvp-CPCM (C_6H_6) level of theory, and Gibbs free energies (G) in Hartree of the structures was calculated by adding up E and TCG. 9

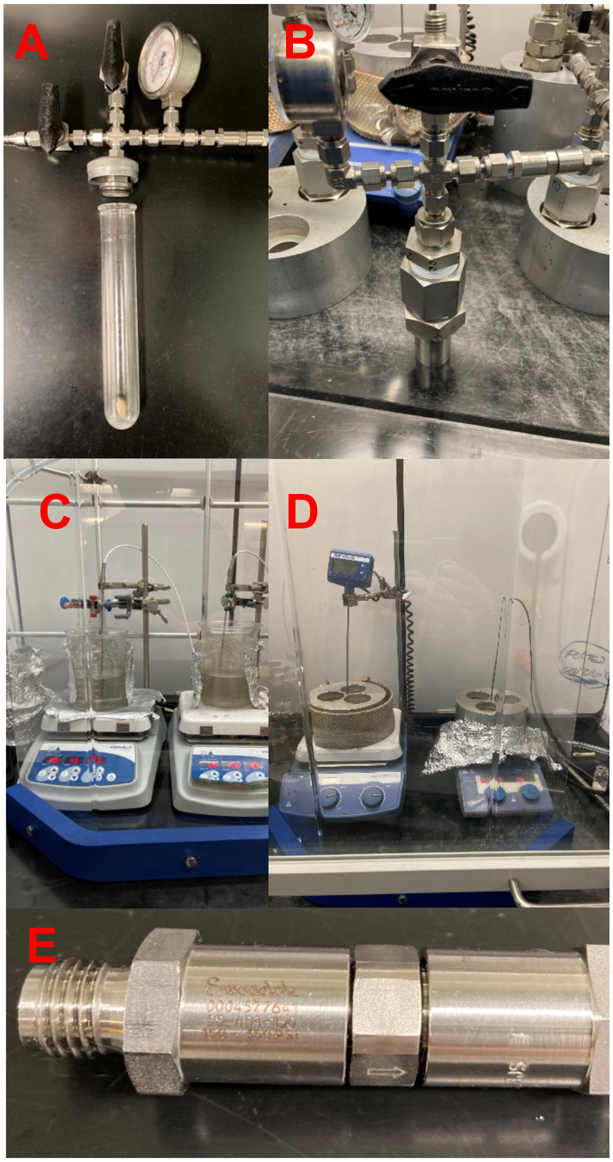

Figure S1. Photographs of: A) 3 oz Fisher-Porter reactor with reactor top fitted with adjustable pressure poppet check valve and pressure gauge, B) stainless steel reactor fitted with adjustable pressure poppet check valve and pressure gauge, C) two oil baths behind a blast shield, D) two heated aluminum blocks behind blast shield, and E) close-up of adjustable pressure poppet check valve.

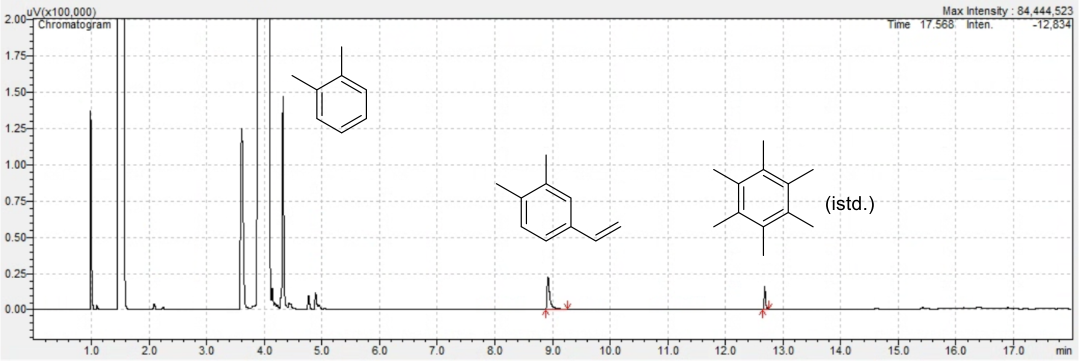

Figure S2. Representative GC-FID chromatogram for o-xylene ethenylation.

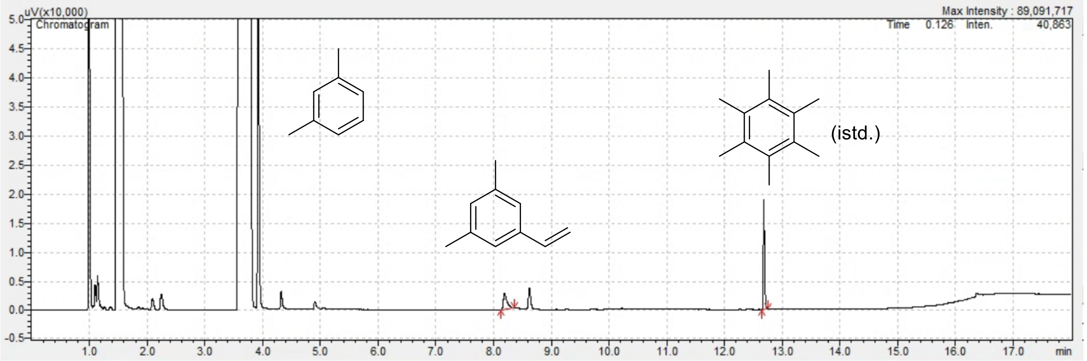

Figure S3. Representative GC-FID chromatogram for *m*-xylene ethenylation.

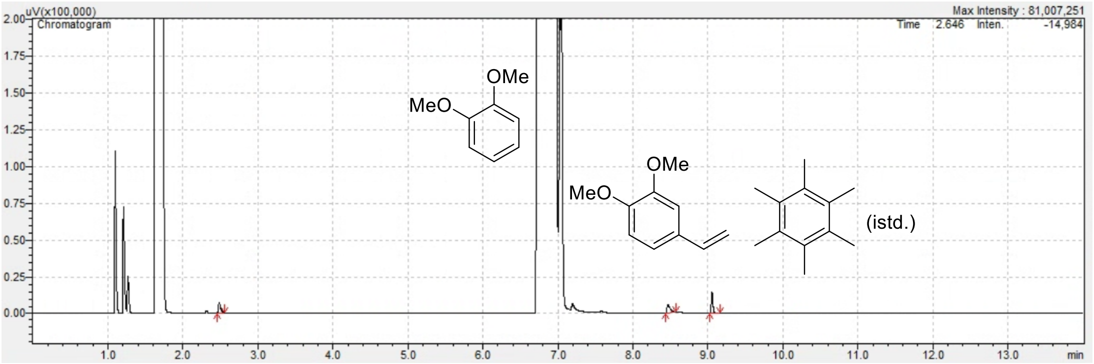

Figure S4. Representative GC-FID chromatogram for 1,2-dimethoxybenzene ethenylation.

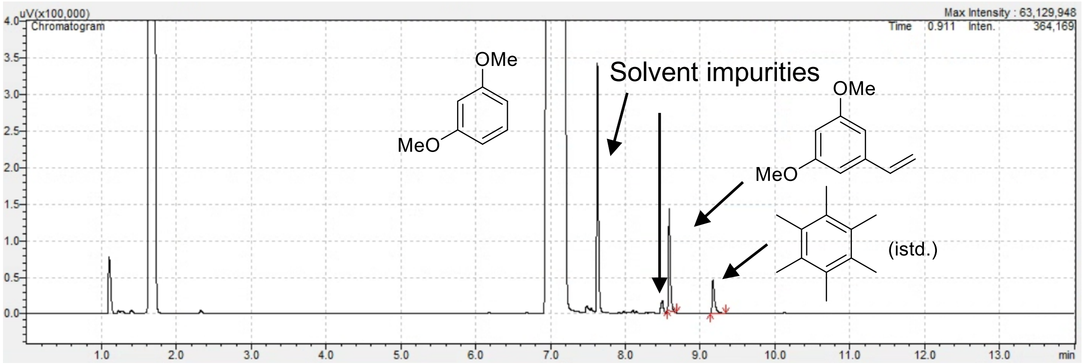

Figure S5. Representative GC-FID chromatogram for 1,3-dimethoxybenzene ethenylation.

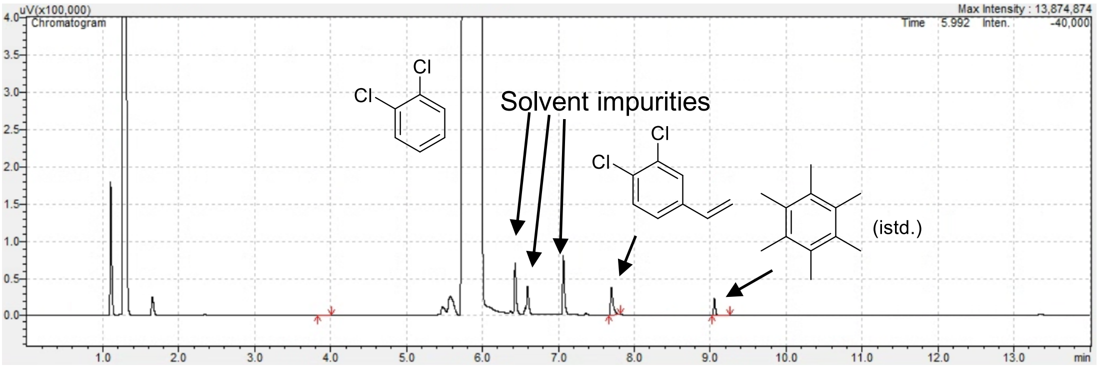

Figure S6. Representative GC-FID chromatogram for 1,2-dichlorobenzene ethenylation.

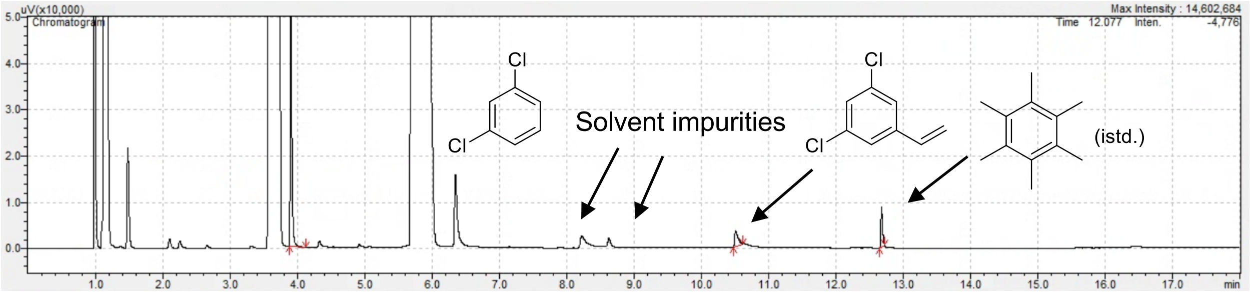

Figure S7. Representative GC-FID chromatogram for 1,3-dichlorobenzene ethenylation.

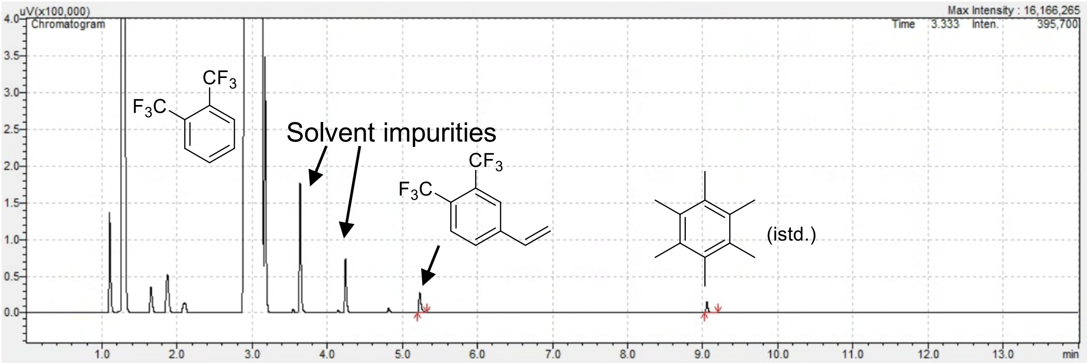

Figure S8. Representative GC-FID chromatogram for 1,2-bis(trifluoromethyl)benzene ethenylation.

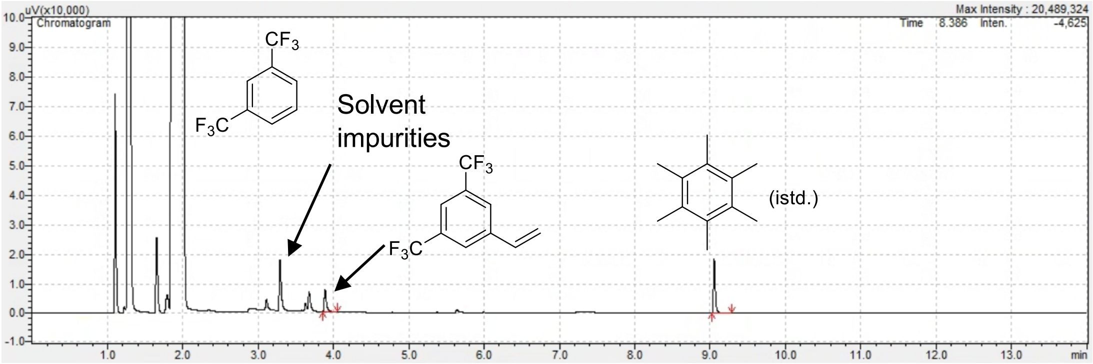

Figure S9. Representative GC-FID chromatogram for 1,3-bis(trifluoromethyl)benzene ethenylation.

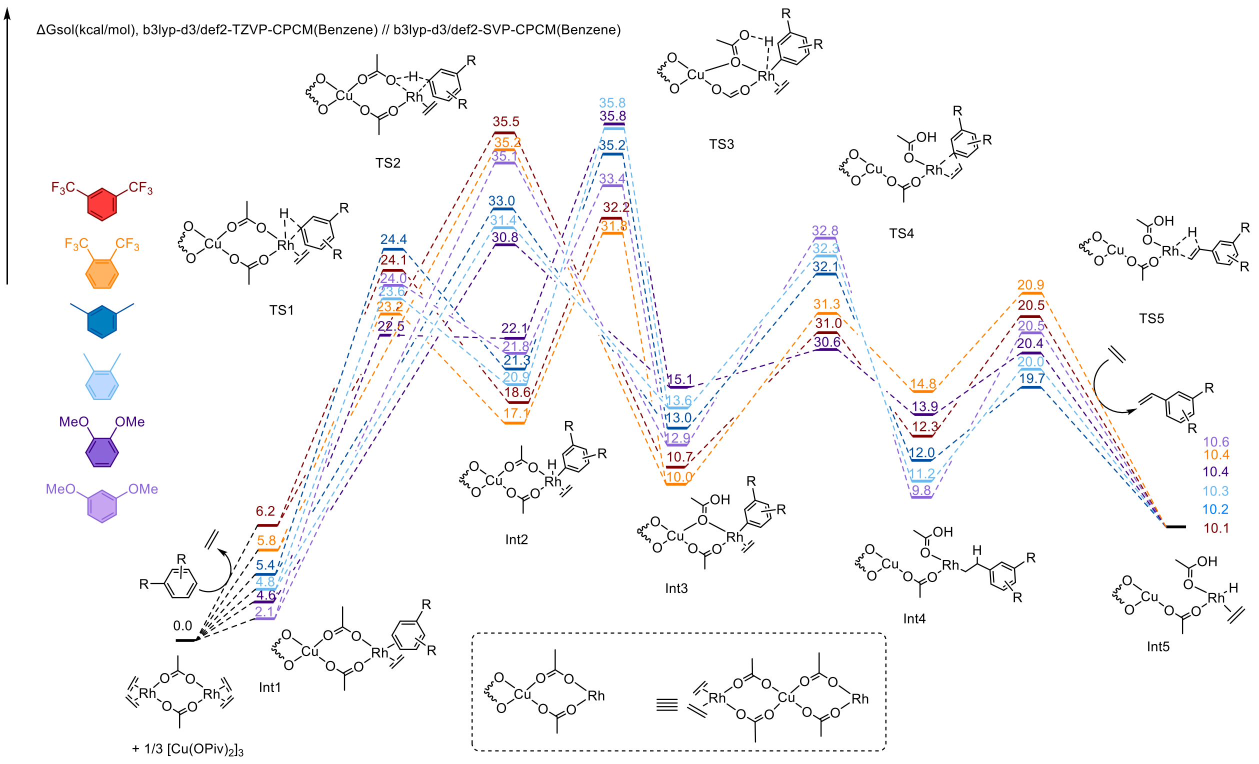

Figure S10. *Energy profiles for all six substrates. DFT-computed free energy changes of substituted styrene formation. Computations are carried out at (U)B3LYP-D3/def2-SVP-CPCM(Benzene) // (U)B3LYP-D3/def2-TZVP-CPCM(Benzene) level of theory. Energies are given in kcal/mol. Mechanisms for different substrates are differentiated by colors: red, 1,3-bis(trifluoromethyl)benzene; orange, 1,2-bis(trifluoromethyl)benzene; dark blue, m-xylene; light blue, o-xylene; dark purple, 1,2-dimethoxybenzene; light purple, 1,3-dimethoxybenzene.*

Table S1. Energies in Figure S10 and thermal correction to Gibbs Free Energy (TCG) was calculated with geometry optimization at the b3lyp-d3/def2-svp-CPCM (C_6H_6) level of theory, and single point energy (E) was calculated at the b3lyp-d3/def2-tzvp-CPCM (C_6H_6) level of theory, and Gibbs free energies (G) in Hartree of the structures was calculated by adding up E and TCG.

Structures		TCG	E	G
Cu(OAc)$_2$	Monomer	0.065372	-2097.81	-2097.74
	Trimer	0.234626	-6293.52	-6293.28
Rh$_2$[(OAc)$_2$(C$_2$H$_4$)$_4$]		0.26728	-993.12	-992.853
Disubstituted Benzene	mCF$_3$	0.070221	-906.739	-906.669
	oCF$_3$	0.072847	-906.731	-906.658
	mMe	0.12087	-311.011	-310.89
	oMe	0.124206	-311.011	-310.887
	mOMe	0.13112	-461.494	-461.363
	oOMe	0.130278	-461.486	-461.356
Ethylene		0.028438	-78.6249	-78.5965
Int1	mCF$_3$	0.399408	-3919.08	-3918.68
	oCF$_3$	0.398884	-3919.06	-3918.67
	mMe	0.449325	-3323.35	-3322.9
	oMe	0.449419	-3323.35	-3322.9
	mOMe	0.45886	-3473.84	-3473.38
	oOMe	0.45621	-3473.82	-3473.37
TS1	mCF$_3$	0.398732	-3919.05	-3918.65
	oCF$_3$	0.400631	-3919.04	-3918.64
	mMe	0.446189	-3323.31	-3322.87
	oMe	0.446906	-3323.31	-3322.87
	mOMe	0.454204	-3473.8	-3473.34
	oOMe	0.45155	-3473.79	-3473.34
Int2	mCF$_3$	0.398232	-3919.05	-3918.66
	oCF$_3$	0.397038	-3919.04	-3918.65
	mMe	0.445515	-3323.32	-3322.87
	oMe	0.447698	-3323.32	-3322.87
	mOMe	0.457886	-3473.8	-3473.35
	oOMe	0.454416	-3473.79	-3473.34
TS2	mCF$_3$	0.394942	-3919.02	-3918.63
	oCF$_3$	0.395026	-3919.01	-3918.62
	mMe	0.445948	-3323.3	-3322.85
	oMe	0.445949	-3323.3	-3322.85
	mOMe	0.455635	-3473.78	-3473.32
	oOMe	0.452199	-3473.77	-3473.32
TS3	mCF$_3$	0.392578	-3919.03	-3918.63
	oCF$_3$	0.392889	-3919.02	-3918.62
	mMe	0.442513	-3323.29	-3322.85
	oMe	0.443646	-3323.29	-3322.85

	**mOMe**	0.449946	-3473.78	-3473.33
	**oOMe**	0.450979	-3473.77	-3473.32
**Int3**	**mCF_3**	0.394237	-3919.06	-3918.67
	**oCF_3**	0.394605	-3919.05	-3918.66
	**mMe**	0.444589	-3323.33	-3322.89
	**oMe**	0.447074	-3323.33	-3322.88
	**mOMe**	0.45604	-3473.82	-3473.36
	**oOMe**	0.455203	-3473.8	-3473.35
**TS4**	**mCF_3**	0.393778	-3919.03	-3918.64
	**oCF_3**	0.397327	-3919.02	-3918.63
	**mMe**	0.444696	-3323.3	-3322.86
	**oMe**	0.447844	-3323.3	-3322.85
	**mOMe**	0.45443	-3473.78	-3473.33
	**oOMe**	0.455002	-3473.78	-3473.32
**Int4**	**mCF_3**	0.398058	-3919.06	-3918.67
	**oCF_3**	0.401829	-3919.05	-3918.65
	**mMe**	0.449756	-3323.34	-3322.89
	**oMe**	0.450414	-3323.34	-3322.89
	**mOMe**	0.456334	-3473.82	-3473.36
	**oOMe**	0.457595	-3473.81	-3473.35
**TS5**	**mCF_3**	0.389481	-3919.04	-3918.65
	**oCF_3**	0.392992	-3919.03	-3918.64
	**mMe**	0.44024	-3323.32	-3322.88
	**oMe**	0.443833	-3323.32	-3322.87
	**mOMe**	0.450718	-3473.8	-3473.35
	**oOMe**	0.449984	-3473.79	-3473.34
**Int5**		0.321264	-3013.51	-3013.19
**Disubstituted Styrene**	**mCF_3**	0.098245	-984.171	-984.073
	**oCF_3**	0.101848	-984.163	-984.062
	**mMe**	0.149632	-388.444	-388.294
	**oMe**	0.153149	-388.444	-388.291
	**mOMe**	0.160016	-538.926	-538.766
	**oOMe**	0.159777	-538.919	-538.759
